# Attitudes of healthcare professionals and researchers toward wearable and app derived patient generated health data

**DOI:** 10.1038/s41746-025-01568-4

**Published:** 2025-03-30

**Authors:** Stefanie Brückner, Olamide Sadare, Sabrina Fesl, Madlen Scheibe, Caroline Lang, Stephen Gilbert

**Affiliations:** 1 Else Kröner Fresenius Center for Digital Health, TUD Dresden University of Technology, Fetscherstraße 74, Dresden, Germany; 2 Center for Evidence-Based Healthcare, Faculty of Medicine and University Hospital Carl Gustav Carus, TUD Dresden University of Technology, Fetscherstraße 74, Dresden, Germany

**Keywords:** Health care, Medical research

## Abstract

Patient-generated health data (PGHD) from apps and wearables hold significant potential for enhancing personalised care and medical research. Healthcare professionals (HCPs) are key to its successful adoption, as their attitudes can either support or hinder its integration into clinical practice. This review systematically analysed studies on HCPs’ and researchers’ perspectives on PGHD for clinical and research use. Three databases were searched for articles published between January 2013 and April 2023. Of 246 articles screened, 33 met the inclusion criteria. While most participants viewed PGHD positively, concerns around data security, reliability, and workflow integration persist. Addressing these barriers is essential to maximising PGHD’s benefits for participatory medicine and improved clinical outcomes. The included studies presented medium methodological quality, particularly among quantitative and mixed methods with risks of sampling and nonresponse bias, and often low sample sizes in qualitative studies. However, recurring themes across studies allow a valuable interpretation of the findings.

## Introduction

If you are a physician or allied healthcare professional (HCP), you will likely have had patients present you with printouts of their app-generated health data reports or show you heart rate measurements on their smartwatches, often during time-pressured consultations. The rise of consumer health technologies, such as smartphone health apps and wearables with sophisticated sensors, has led to a new category of medical information: patient-generated health data (PGHD). In contrast to classic clinical data that is collected in clinical settings and interactions with care providers, PGHD are captured outside clinical care settings by patients^1^. Consumer health devices allow citizens to track various health and wellness parameters throughout their daily activities, including vital signs, lifestyle information and quality of life data^1^. Interpretive analysis of this data and actionable guidance holds the promise to support individuals in managing their own health^2^. The value of PGHD extends beyond individual patient care (primary use), as evidenced by the growing interest of researchers, policymakers and related stakeholders in using this data for secondary, population-benefit use cases such as medical research^3^. Digital infrastructures for a safe and secure exchange of digital health data are a prerequisite for its use and focus of many countries on their way to data-driven, digitalised healthcare systems. The recent EU policy initiative of the European Health Data Space (EHDS) is an example of this movement^4^. The EHDS aims to establish a data exchange platform for clinical data and PGHD across EU member states for primary and secondary use^4^.

Given the significant potential of PGHD in patient care and research, it is crucial to understand the attitudes and experiences of HCPs and research staff regarding its use. Several reviews have been conducted summarising research evidence in various areas of PGHD use, care impact and stakeholder opinions. One review looked at PGHD to measure real-world clinical outcomes and found that various PGHD types are currently explored in various disease indications, but their actual impact on health outcomes remains open^5^. Previous reviews have investigated the role of PGHD in clinical decision-making^6^ and the effect on the patient-provider relationship^7^. Our review complements existing literature by summarising opinions from HCPs and researchers from broad professional backgrounds and diverse clinical care settings towards using PGHD from apps and wearables for primary and secondary use. This includes anticipated benefits in care provision, patient management and clinical workflow. It further investigates barriers and concerns about using PGHD of the respective stakeholder groups.

## Results

### Search results

A total of 299 records were retrieved from the electronic databases PubMed, Embase, and Google Scholar (Table 1). After removing duplicates, 246 articles remained for the title-abstract screening, of which 25 were included for the full-text screening. Of these, 15 met the inclusion criteria^8–22^. Six additional articles were identified through a hand search on Google^23–28^ and 12 through reference tracking of included studies (forward tracking^29–32^ and backward tracking^33–40^). A total of 33 studies were included in the thematic analysis^8–40^. Title–abstract screening and full-text screening reasons for exclusion are summarised in Supplementary Information Table 2.

**Table 1:**
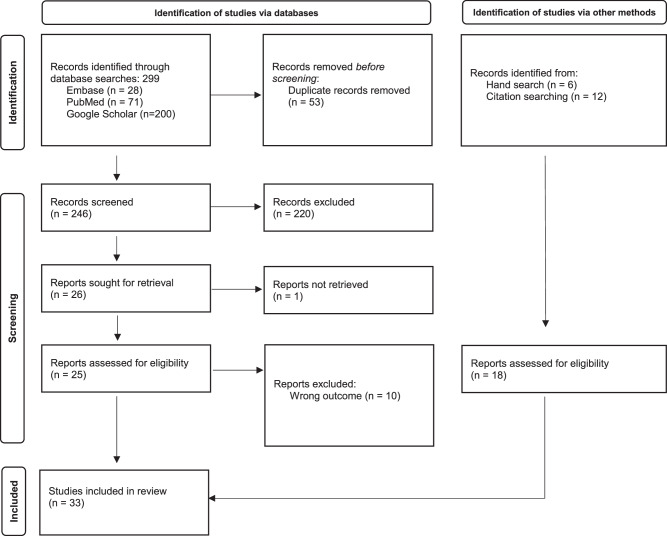
Preferred Reporting Items for Systematic Reviews and Meta-Analyses (PRISMA) flow diagram for literature search and selection process

### Characteristics of included studies and quality assessment

Table 2 summarises the key characteristics of the included studies. The complete data extraction including recruitment information, non-healthcare professional participant groups, data collection and analysis can be found in Supplementary Information Table 3. Of the 33 studies, 24 had a qualitative^8–11,14,16–22,24,26–28,30,31,33,35,36,38–40^, six a quantitative^12,13,15,25,29,34^ and three studies had a mixed methods study design^23,32,37^. Most qualitative studies (19/24) were based on interviews^9–11,14,16,18–22,24,26–28,31,33,38–40^, and the rest (5/24) used focus groups^17,35^, group discussions^8,30^ or a card-sorting session^36^. Mixed methods studies combined surveys with interviews (2/3)^23,37^ or focus groups (1/3)^32^. All quantitative studies were based on surveys for data collection^12,13,15,25,29,34^.

**Table 2:** Characteristics of included studies

Author (year)	Country	Objective	Study design	Medical professions (number of participants)	Disease indication focus	mHealth technology	Main findings
Abdolkhani et al. ^8^	AUS	To identify challenges regarding PGHD quality and potential solutions from the perspective of consumer, healthcare provider and app manufacturer	Qualitative	Clinicians (5)	Not specified	Wearables (consumer technology and medical grade)	HCPs identified various challenges regarding PGHD quality that need to be addressed to ensure its use. They view collaboration with patients on PGHD tracking and use (remote monitoring) as a partnership in which HCPs should be the trusted first point of decision-making for patient care. Manufacturers of these tools should aim to collect clinical evidence and design tools aligned with the standards of healthcare settings.
Adler-Milstein & Nong^33^	USA	To characterize provider-led PGHD approaches, assess their alignment with patient preferences, and identify challenges to scaling and impact	Qualitative	Representatives of health systems (6)	Not specified	Various mHealth technologies, including patient’s own devices, in-clinic tablets, and health system-provided devices	Health system representatives identified three PGHD categories currently pursued: health history, validated questionnaires/surveys, and biometrics/activity (e.g., blood pressure). They also identified barriers to PGHD adoption: data value and quality, lack of reimbursement, and lack of workflow integration.
Andrews et al. ^29^	UK	To explore the views of healthcare providers on remote monitoring through health apps and wearables for epilepsy, multiple sclerosis and depression	Quantitative	HCPs (1006): Doctor (excl. GPs) (138), GPs (118), research/healthcare science (24), management (40), nursing (268), pharmacy (15), psychological professions (157), student (10), wider healthcare team (76), not clear (48)	Epilepsy, multiple sclerosis and depression	Health apps and wearables for remote measuring/ monitoring	HCPs were positive about using PGHD for remote monitoring. Various PGHD types were considered helpful depending on the indication. PGHD should be accessible to the care teams before, during, and after consultations. Concerns were workload, increasing patient anxiety, and reinforcing health disparities.
Austin et al. ^9^	UK	To evaluate a rheumatoid arthritis (RA) PGHD monitoring system for acceptability and feasibility (within REMORA study setting)	Qualitative	Clinicians (2)	Rheumatoid arthritis	REMORA smartphone app for patients with an EHR/research database integration	Clinicians appreciated that the collected PGHD provided a *bigger picture*, identifying real-time changes in disease activity and capturing symptoms that would otherwise have been missed. PGHD can be used to set an agenda for a consultation and to support patients’ memories.
Berkowitz et al. ^10^	USA	To investigate healthcare provider perspectives on the opportunities and barriers for mHealth app use in oncology care	Qualitative	Oncology care professionals (15): Physicians (8), advanced practice providers, e.g. nurse practitioners, physician assistants (3), supportive service providers, e.g. social workers, nursing support staff (4)	Cancer	Health apps for cancer care	HCPs report limited exposure to oncology apps in patient care but were generally open to using them. Expected benefits are health promotion, symptom tracking and patient engagement. Perceived barriers are access to technology, responsibility, workflow disruption and the source of the app itself.
Bietz et al. ^23^	USA	To understand experiences, utility and barriers to using personal health data in research from the perspectives of early adopters, researchers, and companies with experience in PGHD	Mixed methods	Researchers (134): Health Science, social science, life science, engineering and technology, arts and humanities	Not specified	Health apps and wearables (consumer technology)	Most researchers (89%) perceive PGHD as useful, especially vital signs, stress levels, and mood. Almost all researchers believe PGHD can answer questions traditional clinical data cannot. However, there are barriers to using PGHD, including data quality and validity, IP concerns, licensing, collaboration with companies, and others.
Bruno et al. ^34^	UK	To investigate the perspectives of people with epilepsy, caregivers, and healthcare professionals on the current use of and willingness to use digital technology and wearables for seizure monitoring	Quantitative	HCPs (22): Neurologists (10), psychiatrist (1), epilepsy nurses (7), service managers (2), disability nurse (1), medical student (1)	Epilepsy	Health apps and wearables for remote monitoring	HCPs see the usefulness of digital tools for patient management (68.2%), especially for specific decision points (e.g., treatment adjustments); however, 40% state that this information is never or rarely used. Half of HCPs are concerned about increased workload due to data reviewing and suggest a nurse as an appropriate team member to deal with PGHD.
Cohen et al. ^11^	USA	To examine the experiences of HCPs in outpatient clinics using PGHD in care as part of a national research initiative (Project HealthDesign programme)	Qualitative	HCP (25): Physicians, nurses, health coaches (12); study team members (13)	Chronic conditions (asthma, cognitive decline, overweight, Crohn’s disease)	Consumer mHealth technologies	HCPs see benefits in PGHD access in three main areas: deeper insights into patients, more accurate patient information, and insights between clinical visits. PGHD implementation requires adaptation in clinical workflows and patient-provider alignment on communication expectations (when used in remote monitoring settings).
Gabriels & Moerenhout^40^	BE	To analyse how physicians evaluate classic and digital self-tracking in everyday clinical practice and to explore the impact of digital self-tracking on self-care and professional healthcare	Qualitative	Physicians (12): GPs (7), cardiologists (5)	Not specified	Health apps and wearables (consumer technology)	GPs and cardiologists had little experience with PGHD from consumer technologies, and it’s not yet an integrated part of their clinical practice. The interpretation of this data, data overload, and the potential of an emerging “entertainment medicine” were concerns.
Haase et al. ^24^	DK	To investigate GPs engagement with patient-provided data from new technologies	Qualitative	GPs (23)	Not specified	Wearables (consumer technology), online symptom checker (commercial company or health system provided)	Only a few patients bring PGHD to a consultation (sleep data, heart rate data or symptom checker). Those PGHD are not analysed as measurements but more as another symptom description from the patient. Patient-initiated data generation was considered less relevant in comparison to healthcare system generated data - even if the underlying test is the same.
Huh et al. ^35^	USA	To understand different healthcare professionals’ and patients’ perspectives about the use of self-monitoring tools for older adults’ personal wellness	Qualitative	HCP (10): nursing experience (8), director of a nursing facility (1), geriatric psychiatrist (1)	Geriatric care	Health apps and wearables (consumer technology)	HCPs were positive about wellness-related PGHD from self-monitoring tools. They found benefits in improving patient-provider communication and educating patients and caregivers. HCPs assumed they controlled selecting PGHD for tracking and what/when to present it to the patient.
Jacomet et al. ^12^	FR	To address whether mHealth serves people living with HIV and their physicians as part of a broader self-managed care service	Quantitative	HIV care physicians (255)	HIV infection	Health apps and wearables (consumer technologies)	Health apps are not yet integrated into standard HIV care. However, HCPs see the potential, including in derived PGHD: 40% see apps as helpful for clinical decision support and 37% for monitoring improvements. Only 18% thought apps could improve patient-provider relationships.
Karduck & Chapman-Novakofski^13^	CA	To identify factors that may be associated with app use by clinicians working in diabetes and weight management	Quantitative	Clinicians (719): Registered dietitian nutritionists, registered nurses, certified diabetes educators, board-certified advanced diabetes, advanced practice nurses, doctorates of pharmacy	Diabetes, obesity	Health apps and wearables (consumer technologies)	Most clinicians (62%) recommended smartphone apps to their clients to track diet and physical activity levels. More than 80% of clinicians preferred digital tracking with apps over traditional methods. Discussed barriers include patients’ literacy, inaccurate data through app errors and workload on patient.
Kelley et al. ^36^	USA	To understand the perspectives of student health professionals on the usefulness of tracking for assessment, communication and self-care planning for student’s mental wellbeing	Qualitative	Student Health Professionals (14): Psychiatry (9), primary care (2) women’s health (2), health promotion (1)	Mental Health	Health apps and wearables (consumer technology)	Student health professionals perceive access to PGHD as useful depending on data type and case context.
Keogh et al. ^14^	IRE	To understand the experiences and opinions of researchers from academic, industry and clinical contexts in the use of wearable devices to measure gait and physical activity	Qualitative	Researchers (20): biomedical science (2), computer science (1), doctor (4), engineering (3), information technology (1), physiology and/or sport and movement science (4), physiotherapy (5)	Mobility issues	Wearables (consumer technology and medical grade)	Researchers value PGHD from wearables because of its novel insights, which complement traditional data sets. PGHD use requires new protocols. Barriers to use include data management and clear clinical utility.
Kessel et al. ^25^	DE	To investigate the attitude HCPs toward telemedicine, mHealth, and mobile apps in oncology	Quantitative	Internal medicine, surgery and other care professionals (108): Resident physicians (24), attending physicians (17), senior physicians (27), heads of department (8), nurses (15), others (17)	Cancer	Health apps for cancer care	Most HCPs (84.3%) supported the idea of an oncological app to complement classical treatment, supporting consultation and patient-provider communication with PGHD. Listed helpful features for PGHD collection included side effects, quality of life and others. Most HCPs (93.5%) also supported the use of collected data for scientific research.
Kim et al. ^32^	CA	To explore clinicians and older adults’ perceptions of PGHD	Mixed methods	HCPs (4): primary care physician (1), nurses (2), physiotherapist (1)	Geriatric care	Health apps and wearables (consumer technology)	Clinicians evaluated PGHD as useful for monitoring treatment and identifying trends/triggers for older adults. Data reliability, e.g. through noncompliance, was a concern, as well as data privacy/security issues, workload and data overload. Identified useful PGHD included blood glucose, step count, physical activity, sleep, blood pressure, and stress level.
Kong et al. ^15^	USA	To investigate physicians’ attitudes towards the adoption of mHealth technologies	Quantitative	Physicians of 36 medical specialities (186)	Not specified	Health apps and wearables (consumer technology)	A majority of physicians see collected biometrics from apps and wearables as useful to promote a healthy lifestyle (68%), track medical treatment (64%), or conduct research (56%). Proof of accuracy and precision (81%) - and the efficient integration of collected data (68%) - preferably directly in EHR - were identified as important improvements.
Lavallee et al. ^16^	USA	To investigate HCPs’ perspectives and experiences in PGHD use to understand associated value and barriers	Qualitative	HCPs (15)	Not specified	Health apps, wearables and geolocation technologies (consumer technologies)	HCPs see many benefits of using PGHD along the patient journey, including supporting care decisions and improving patient-provider communication and engagement. Barriers to using PGHD are concerns about data validity and lack of integration in clinical workflow.
Nguyen et al. ^26^	AUS	To investigate GPs perspectives of their current and future roles in the use of health apps by their patients and how patient-focused apps affect patient management	Qualitative	GPs (10)	Not specified	Health apps (consumer technology	GPs see the benefit of health apps in patient care and PGHD as an additional source of information about a patient. However, apps and PGHD are not yet integrated in clinical practice.
Nundy et al. ^37^	USA	To explore HCP perceptions of a PGHD report from a text-message-based diabetes self-management programme	Mixed methods	Primary care physicians and endocrinologists (12)	Diabetes	App for text-based diabetes monitoring	Only 25% of HCPs felt access to PGHD diabetes report impacted the care they provided. However, 75% would be willing to continue using it. Perceived benefits of PGHD included agenda setting, assessment of self-care, and identification of patient barriers. Concerns were raised about which patients should track and what, data reliability and workflow integration.
Osborne et al. ^17^	USA	To identify app content and feature needs from individuals with stroke and traumatic brain injury, caregiver and care provider	Qualitative	Neurorehabilitation therapists (8)	Stroke and traumatic brain injury	Health apps	Therapists favour an app for remote access to PGHD with data integration into the EHR to allow collaboration among HCPs. To manage workload, a dedicated care coordinator should review data first and alert other HCPs accordingly.
Ostherr et al. ^27^	USA	To investigate why and how researchers, health technology start-up companies, and members of the general public interact with and understand the value of PGHD	Qualitative	Behavioural and computational scientists (10)	Not specified	Health apps and wearables (consumer technology)	Research reported difficulties recruiting patients for research that involves them sharing PGHD - which is in great contrast to the finding that members of the general public who are using wearables and/or health apps expressed little concern about sharing health data with the companies that provide the devices or apps. Researchers have concerns about data interpretation and trust in the source of PGHD.
Reading et al. ^38^	USA	To investigate individual patient differences in sustained engagement among individuals with a history of Atrial fibrillation (AF) who are self-monitoring using mHealth technology (iHeart trial)	Qualitative	AF care professionals (8): nurse practitioners (4), physicians (2), research coordinators (2)	Atrial Fibrillation	AliveCor ECG monitor and app	HCPs see the device’s usefulness for patient self-management and medical care. Interaction and feedback from HCPs on PGHD impacted patients’ engagement status. HCPs expressed concerns about additional workload and unaligned expectations regarding feedback and tracking burdens for patients.
Saleem et al. ^28^	USA	To assess clinicians’ perspective on the use of Fitbit PGHD to care for their Veteran patients and sharing data with the U.S. Department for Veteran Affairs (Fitbit pilot programme).	Qualitative	Veteran care professionals (16): dieticians (7), physical therapists (2), physicians (2), nurse practitioner (1), sleep medical technologist (1), respiratory therapist (1), nurse (1), licensed practical nurse (1)	Not specified	Fitbit, data sync app	Veteran clinicians saw the benefit of having Veterans use Fitbits and saw the value of PGHD in the Veterans’ care plan, including monitoring progress towards health behaviour goals.
Sanger et al. ^18^	USA	To investigate the tensions between patients’ and providers’ needs when designing a novel, patient-centred technology – mobile Post-Operative Wound Evaluator (mPOWEr) – that uses PGHD for post-discharge surgical wound monitoring.	Qualitative	Surgery care professionals (11): Attending physician (4), resident physician (1), nurse practitioners (3), physician assistant (1), clinic nurses (2)	Post-discharge surgical site infection	mobile Post-Operative Wound Evaluator app (mPOWEr)	HCPs and patients recognise PGHD as useful in acute, post-surgical care settings. However, disagreements about data collection and feedback expectations cause tensions.
Sarradon-Eck et al. ^19^	FR	To investigate GPs’ perception and expectations toward prescription or recommendation of patient-focused mHealth apps or devices	Qualitative	GPs (36)	Not specified	Health apps and wearables (consumer technologies and on prescription)	GPs see health apps as tools to engage patients in their health management. While PGHD are considered valuable as an additional longitudinal data source, the extra workload created by reviewing data not integrated into the EHR and resulting medical liability questions are of concern. GPs were also concerned about overmedicalisation, de-humanisation of the patient-doctor relationship and commodification of patient data.
Volpato et al. ^30^	CH	To explore GPs’ perceptions of the role, benefits, risks, challenges, and future development of wearable devices in family medicine	Qualitative	GPs (19)	Not specified	Health apps and wearables (consumer technology and medical grade)	GPs were positive about using wearables/apps for remote monitoring (epilepsy and cardiac diseases as examples), supporting self-management and health goals, as well as research. Concerns were related to PGHD quality and validity, lacking clinical evidence for devices, data privacy and security issues, and data workload.
Watt et al. ^31^	UK	To explore HCPs’ attitudes toward their patients’ use of wearable technology	Qualitative	HCPs (12): (GPs (4), junior doctors (3), dietician (1), personal trainer/pharma-ceutical technician (1), consultant nurse (1), occupational therapist (1), and physiotherapist (1)	Not specified	Wearables (consumer technology)	HCPs saw value in wearables for self-management that could lead to health improvements and reduced costs for the health systems. Concerns were raised about health obsession, distress through tracking and also the intrusion of the patient’s most private sphere by accessing PGHD. Another question was who should pay for the devices if patients are supposed to use them.
Wendrich & Krabbenborg^20^	NL	To investigate HCPs’ perspectives on using smartphone apps for digital self-monitoring in multiple sclerosis (MS), particularly focusing on physician-patient communication, healthcare providers respond to self-monitoring data and the role of patient	Qualitative	HCPs (14): Neurologist (4), MS specialist nurses (7), rehabilitation physicians (2), occupational therapists (1)	Multiple sclerosis	Health apps and wearables MS self-monitoring	MS care specialists were willing to use self-monitoring apps and valued the quantitative data complementing patients’ narratives. HCPs wanted to control what app is used and what PGHDs are tracked while delegating tasks to patients. Concerns about the workload on patients and emotional burden were raised.
West et al. ^39^	UK	To investigate HCP perceived barriers to using PGHD across distinct workflows in clinical settings	Qualitative	HCPs (13): Cardiologists (4), Mental health specialists (2), emergency doctor (1), junior surgeon (1); hospital doctor (1), GP (1), heart failure nurse (1), oncology nurse (1), audiologist (1)	Not specified	Health apps and wearables (consumer technologies)	HCPs were positive about PGHD use depending on their medical speciality (surgeon less than cardiologist). Perceived barriers to the use of PGHD depend on the specific clinical context.
Wu et al. ^21^	USA	To investigate the current use of PGHD within mental health care with a focus on workflow integration, clinicians’ perspectives on PGHD and selection of tools for patients	Qualitative	HCPs (12): Psychiatrists (7), clinical psychologists (5)	Mental health	Health apps and wearables (consumer technologies)	Mental health clinicians reported PGHD collection has always been a part of mental health practice. However, collection and management are not standardised or optimised. PGHD are considered as valuable information, but concerns are raised about data validity, reliability and workflow integration.
Zhu et al. ^22^	USA	To identify enablers and barriers inherent to sharing PGHD for patient-clinician communication and to gain insights into design requirements for future technology interventions	Qualitative	HCPs (9): Physical therapist (1), internists (4), primary care physician (1), psychologist (1), paediatric nephrologist (2)	Not specified	Health apps and wearables (consumer technologies)	Various technical, social, and organisational challenges were discussed for using PGHD in clinical practice, including sharing approaches, reimbursement, expectation management, quality of PGHD, and workload.

*AF* Atrial fibrillation, *AUS* Australia, BE Belgium, *CA* Canada, *CH* Switzerland, *DE* Germany, *DK* Denmark, *EHR* Electronic health record FR France, *GP* General practitioner, *HCP* Healthcare professional, *IRE* Ireland, *MS* multiple sclerosis, *NL* Netherlands, *PGHD* patient-generated health data, *RA* rheumatoid arthritis, *UK* United Kingdom.

Most studies (22/33) recruited a mix of healthcare professionals for their data collection, including physicians, psychologists, therapists, and nurses among others^8–11,13,16–18,20–22,25,28,31–34,38,39^. Eight studies focused on physicians only^12,15,19,24,26,30,37,40^. Three studies specifically focused on the perspectives of researchers and will be separately analysed and can be found in the Supplementary Information Note 3^14,23,27^.

Fifteen studies explored opinions toward health apps and/or wearables generally^8,15,16,19,22–24,26–28,30,31,33,39,40^, and 18 focused on a specific disease indication^9–14,17,18,20,21,25,29,32,34–38^, such as mental health conditions^21,36^ or cancer^10,25^.

Six studies^9,11,18,28,37,38^ investigated perspectives of HCPs within a clinical trial/research programme piloting apps and wearables for PGHD collection for patient care (Table 3). One study used a medical device app to monitor ECG in patients with Atrial Fibrillation^38^. Another study investigated a prototype medical device to monitor diabetes and two studies explored non-medical device data handling apps to track symptoms of rheumatoid arthritis based on validated questionnaires^9^ and an image-based wound healing tracker for post-discharge surgical site infection^18^. HCPs in those studies accessed the shared PGHD through web dashboards/portals^18,38^ or reports within^9^ or separate from the EHR^37^. Another study by the U.S. Department of Veterans Affairs piloted the fitness and activity tracker Fitbits for veterans that synced the PGHD via a data sync app to a web-based provider dashboard^28^. Another study interviewed HCPs from five different studies with focus on PGHD sharing. In these studies, PGHD were collected using various, unspecified consumer mHealth devices, with provider access either facilitated through a special dashboard/platform or at the patient’s discretion, e.g., during consultation on their device^11^. In 24 studies, the opinions on PGHD were assessed without actually sharing PGHD; of those, 23 did not differentiate the type of device for data collection.

**Table 3:** Overview on device type for PGHD collection, PGHD types and workflow integration

Device type	No. of studies	Indication focus (disease)	PGHD type shared or discussed	PGHD sharing part of study	Workflow of data sharing between patient and HCP (as part of study or discussed)
Medical device app/wearable	1^38^	Atrial Fibriliation	ECG data^38^	Yes	From patient smartphone to web-based provider portal
Prototype medical device app	1^37^	Diabetes^37^	Glucose measures, medication adherence, programme progress, reported barriers to self-care^37^	Yes	Report shared with HCPs by study staff prior consultation, not further specified^37^
Non-medical device data handling app	2^9,18^	Rheumatoid arthritis^9^	Symptom and disease impact tracking, based on validated questionnaires^9^	Yes	Graphical summaries of longitudinal data in EHR^9^
		Post-discharge surgical site infection^18^	Symptom tracking, wound photos^18^	Yes	Data send to web-based provider dashboard^18^
Wellness & lifestyle app/wearable	2^28,35^	Not specified^28^	Steps, sleep, heart rate, calories burned, exercise/workouts, water consumption, nutrition, breathing, oxygen levels, weight, mindfulness exercises^28^	Yes	Fitbit data automatically sent to web-based provider platform^28^
		Geriatric care^35^	Social, spiritual, cognitive and physiological measures (unspecified)^35^	No	PGHD sharing was not part of study and not discussed^35^
Not specified (Medical device and/or wellness/lifestyle app/ wearable)	24	Chronic conditions (various)^11^	Not specified	Yes	Data from patient device to web-based provider dashboard^11^ or through patients choice^11^
		Epilepsy^29,34^	Breathing rate, sweating^29,34^ Sleep, heart rate, seizure tracking, body movements, voice quality, environmental factors, mood, medication/therapy adherence, concentration^34^ Skin/body temperature^29^	No	Integration in EHR or presentation on patient’s device desired^29,34^
		Multiple Sclerosis^20,29^	Breathing rate, skin/body temperature, voice quality	No	Integration in EHR^20,29^ or presentation on patient’s device desired^29^15/03/2025 13:47:00
		Mental Health^21,29,36^	Sleep^21,29,36^ Environmental information^29,36^ Mood, PROMs (e.g., Quality of life)^21^ Body movements, heart rate, breathing rate, smartphone usage, smartphone usage^29^ Behaviour data, nutrition information, weight, BMI, substance use, body image^36^	No	Integration in EHR^21,29^ or presentation on patient’s device desired;^29^ or not specified^36^
		Cancer^10,25^	Symptom tracking, physical activity, nutrition, medication/therapy adherence^10^ Quality of life (PROMs), side effects, treatment satisfaction^25^	No	Integration in EHR desired^10,25^
		Diabetes^13^	Blood glucose measures, physical activity, nutrition, weight, medication/therapy adherence^13^	No	Not specified
		HIV infection^12^	Not specified	No	Not specified
		Stroke and traumatic brain injury^17^	Physical activity, behaviour data^17^	No	Integration in EHR desired^17^
		Geriatric care^32^	Not specified^32^	No	Access via decision support systems desired^32^
		Not specified^8,15,16,19,22,24,26,30,31,33,39,40^	Sleep, heart rate, steps^24,32^ Sweating, skin/body temperature, physical activity, gait, sedentariness, Environmental factors, smartphone usage, communication, social media usage, typing pattern, GPS, blood glucose measures, mood, nutrition, weight, body fat percentage, inhaler use, wound pictures, blood pressure, ECG data, peak expiratory flow^32^15/03/2025 13:47:00	No	Integration in EHR or patient portal desired^8,15,16,19,22,33,39,40^ View on patient device or verbal description^22,24,33,39^ Not specified^26,30,31^

(BMI Body Mass Index; EHR electronic health record; HCP Healthcare professional; PGHD patient-generated health data; PROM patient-reported outcome measure).

Table 4 summarises the detailed quality assessment of all included studies. All studies passed the initial screening questions “Are there clear research questions?” and “Do the collected data allow to address the research question?”. The methodological quality of the included studies was medium. The most frequently found issue in studies with quantitative methods was the risk of sampling bias and non-response bias^12,13,15,23,25,29,32,34,37^. The most frequent issue with qualitative methods in studies was the underreporting of quotes to prove findings. Two studies stood out because of their very low number of HCP participants of five^8,16,17,23,27,30^ or two^9^, respectively.

**Table 4:** Quality evaluation of included studies using the Mixed Methods Appraisal Tool, 2018 version^56^

Authors	Qualitative					Quantitative					Mixed methods					Comment
	1.1	1.2	1.3	1.4	1.5	4.1	4.2	4.3	4.4	4.5	5.1	5.2	5.3	5.4	5.5	
Abdolkhani et al. ^8^	Y	Y	C	C	C											Findings not well backed by quotes; small sample size; sampling bias; response bias risk (personal contacts)
Adler-Milstein & Nong^33^	Y	Y	Y	Y	Y											Small sample size; authors acknowledge potential confusion of PGHD, PRO and Remote monitoring by participants which might impact the quality of the responses
Andrews et al. ^29^						Y	N	C	N	Y						Large sample size; risk of sampling bias towards people with positive view on technology; completion rate not reported
Austin et al. ^9^	Y	Y	Y	Y	Y											Findings well supported by quotes; small number of clinicians
Berkowitz et al. ^10^	Y	Y	Y	Y	Y											Findings well supported by quotes; risk of selection bias towards participants with positive view on technology
Bietz et al. ^23^	Y	Y	C	N	C	Y	C	Y	N	Y	Y	N	C	C	N	Findings are not backed by sufficient quotes which make it difficult to assess the quality of the interpretation and integration with quantitative findings; risk of nonresponse bias (response rate is not reported)
Bruno et al. ^34^						Y	N	C	C	Y						Limitations in sample size and representativeness due to low response rate (40.8%), female gender bias in individuals and care giver group, sampling bias towards people with technology affinity (online portals were used for recruitment); only limited explanation on questionnaire validation
Cohen et al. ^11^	Y	Y	Y	Y	Y											Findings are well supported by quotes and compared across the five different studies
Gabriels & Moerenhout^40^	Y	Y	Y	Y	Y											Findings are well supported by quotes; study provides detailed information on interview guide development and data analysis; small sample size
Haase et al. ^24^	Y	Y	Y	Y	Y											Findings are well supported by quotes; method section of paper already contains results; results section not clearly labelled; risk of response bias (28,75% response rate)
Huh et al. ^35^	Y	Y	Y	Y	Y											Findings are well supported by quotes, low number of HCP participants, sampling bias for older adults (recruited from a community with high overall education level); only one author performed the analysis
Jacomet et al. ^12^						Y	C	C	N	Y						Information on physicians (e.g., professions, years of experience) missing, nonresponse bias for HIV patients (response rate 59%, gender difference in responder vs non-responder); response rate for HCP not specified, information on questionnaire validation missing
Karduck & Chapman-Novakofski^13^						Y	C	Y	N	Y						Large sample size; representativeness of sample unclear, e.g. almost all participants are female but authors don’t describe the expected demographic variables of the target group; despite overall high response rate of 81% there is a risk of non-response bias (difference between responder and non-responder not defined)
Kelley et al. ^36^	Y	Y	C	Y	Y											Findings well supported by quotes; student survey questionnaire without details on validation
Keogh et al. ^14^	Y	Y	Y	Y	Y											Findings are well supported by quotes; divers sample; potential selection bias (participants might have favourable view on wearables because of project involvement)
Kessel et al. ^25^						Y	C	Y	N	Y						Low participation (59.1%) and completion rate (37.2%), risk of nonresponse bias
Kim et al. ^32^	Y	Y	Y	Y	Y	Y	N	N	C	Y	Y	Y	Y	C	N	Small number of participants, bias in sample (only young clinicians participated), non-response bias undiscussed, questionnaire not piloted
Kong et al. ^15^						Y	C	C	N	C						Very low response rate (12.9%), representativeness of sample questionable (target population not specified, low response rate), no information about questionnaire validation, risk of non-response bias
Lavallee et al. ^16^	Y	Y	Y	C	C											Findings are not well backed up by enough quotes; sampling bias through purposive sampling
Nguyen et al. ^26^	Y	Y	Y	Y	Y											Findings were well supported by quotes; small sample size; risk of sampling bias through self-selection of participants; low response rate (10%)
Nundy et al. ^37^	Y	Y	Y	Y	Y	Y	N	C	N	Y	Y	Y	Y	Y	N	Small sample size and drawn from only one medical centre and potential gender bias (75% female), questionnaire validation not described, risk of non-response bias (31 providers contacted but only 11 interviews completed)
Osborne et al. ^17^	Y	Y	Y	C	C											Findings for therapist focus group not backed up by quotes; limited sample size; sampling bias (recruitment of patients from one support group and HCPs from one single clinic)
Ostherr et al. ^27^	Y	Y	Y	C	C											Findings are not supported by enough quotes; risk of sampling bias (even though the response rate for general public participants was 80%); study miss to discuss limitations
Reading et al. ^38^	Y	Y	Y	Y	Y											Small HCP sample size; patient sampling bias (predominantly male, middle- to older-age, and moderately to extremely comfortable with technology)
Saleem et al. ^28^	Y	Y	Y	Y	Y											Risk of sampling bias for already engaged veteran patients and nonresponse bias; frequency of occurrence reported but findings could have been backed up with more original quotes
Sanger et al. ^18^	Y	Y	Y	Y	Y											Findings well supported by quotes; risk of sampling bias (participants from one healthcare system), no representation of dark coloured skin patient participants, limited sample size
Sarradon-Eck etal.^19^	Y	Y	Y	Y	Y											Risk of nonresponse bias for interviews (86.7% nonresponse rate for purposive sampling, snowball sampling not reported), sample overrepresents GPs in training who might be more interested in mHealth through their teaching activities (sampling bias).
Volpato et al. ^30^	Y	C	Y	C	C											Mind-maps are innovative but limited for in-depth analysis and potentially inferior to interviews and other qualitative methods; risk of sampling bias
Watt et al. ^31^	Y	Y	Y	Y	Y											Findings well supported by quotes; limited sample size; risk of sampling bias and response bias as some interviews were personal contacts
Wendrich & Krabbenborg^20^	Y	Y	Y	Y	Y											Findings well supported by quotes; limited sample size; risk of response bias (HCPs might be inclined to report positive views about PGHD as their institutions participate in a pilot study)
West et al. ^39^	Y	Y	Y	Y	Y											Findings of literature review and interviews were integrated; findings well supported by quotes; limited sample size; risk of sampling bias
Wu et al. ^21^	Y	Y	Y	Y	Y											Findings are well supported by quotes; interview and app analyses integrated; limited sample size, potentially sampling bias through convenience sampling
Zhu et al. ^22^	Y	Y	Y	Y	Y											Findings are well supported by quotes; inclusion and exclusion criteria clearly defined, small sample size

What each number corresponds to: 1.1. Is the qualitative approach appropriate to answer the research question? 1.2. Are the qualitative data collection methods adequate to address the research question? 1.3. Are the findings adequately derived from the data? 1.4. Is the interpretation of results sufficiently substantiated by data? 1.5. Is there coherence between qualitative data sources, collection, analysis, and interpretation? 4.1. Is the sampling strategy relevant to address the research question? 4.2. Is the sample representative of the target population? 4.3. Are the measurements appropriate? 4.4. Is the risk of non-response bias low? 4.5. Is the statistical analysis appropriate to answer the research question? 5.1. Is there an adequate rationale for using a mixed methods design to address the research question? 5.2. Are the different components of the study effectively integrated to answer the research question? 5.3. Are the outputs of the integration of qualitative and quantitative components adequately interpreted? 5.4. are divergences and inconsistencies between quantitative and qualitative results adequately addressed? 5.5. Do the different components of the study adhere to the quality criteria of each tradition of the methods involved?

Y=yes. N=no. C=can’t tell (HCP healthcare professional; HIV Human immunodeficiency virus; PGHD patient-generated health data; PRO patient-reported outcome).

### Thematic synthesis

The thematic synthesis yielded a multitude of analytical themes that were grouped into five main categories: 1) Benefits of PGHD for patient care; 2) Improving patient management and clinical workflows; 3) Barriers to use PGHD; 4) Evolving roles of patients and HCPs in a changing healthcare system; and 5) Researchers perspectives on PGHD in medical research. The following sections describe the results for categories one to four. The fifth category is separately analysed and can be found in Supplementary Information Note 3.

### Benefits of PGHD for patient care

**Fig. 1:**
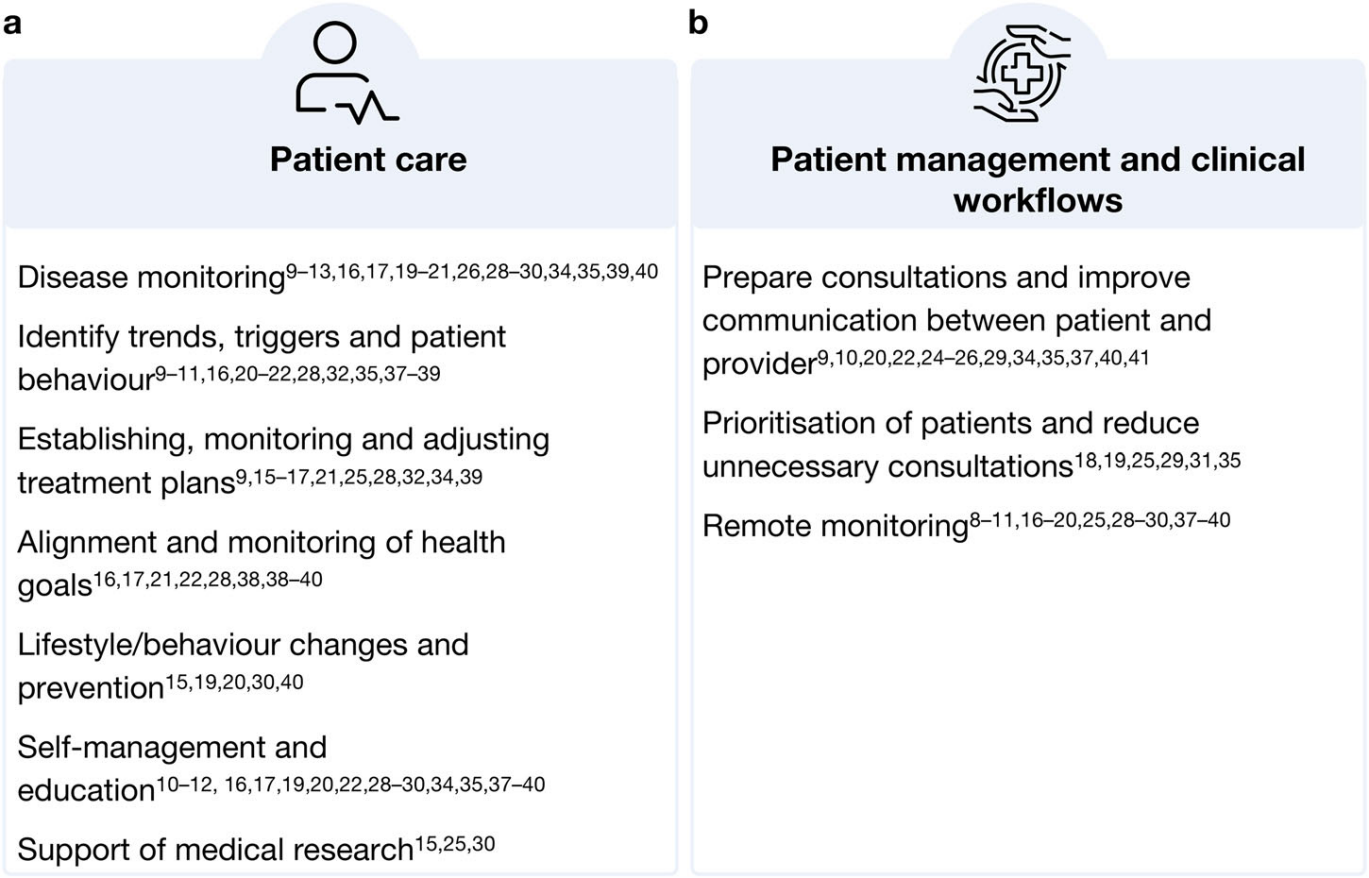
Benefits of PGHD in clinical practice. Synthesised benefits of PGHD in clinical practice identified by HCP participants in included studies. **a** displays benefits in patient care and (**b**) in patient management and clinical workflows. (Author’s summary).

HCPs in various medical specialities and professional groups across studies identified various benefits that access to outside-of-clinic PGHD from health apps and wearables could provide by filling long-existing gaps in the traditional clinical data. Figure 1a summarises the findings. Insights derived from PGHD can support the monitoring and understanding of disease progression and the overall health status (1)^9–13,16,17,19–21,26,28–30,34,35,39,40^. Continuous data collection can help to identify trends, triggers and behaviours impacting patient, health (2)^9–11,16,20–22,28,32,35,37–39^ Further, PGHD can be used to establish, monitor and adjust treatment plans (3)^9,15–17,21,25,28,32,34,39^, enabling collaboration between patient and provider to align and monitor the health goals of patients (4)^16,17,21,22,28,38–40^. PGHD utilisation is further seen as valuable for supporting lifestyle and behaviour changes and promoting prevention (5)^15,19,20,30,40^ and empowering patients to self-manage their diseases (6)^10–12,16,17,19,22,28–30,34,35,37–40^. In addition to its potential in patient care, HCPs in three studies also highlighted PGHD value for medical research (7)^15,25,30^. Two of those studies were quantitative: one with cancer care physicians found that 93% of participants supported using oncology app data for research^25^, and another with physicians from 36 different medical specialities found that 56% saw benefits or medical research^15^. Overall, HCPs found that PGHD utilisation offers significant benefits for both patient care and medical research by providing continuous, personalised health insights and fostering a collaborative approach to health management.

Four studies quantitatively assessed which types of PGHD HCPs considered as useful. For this review, PGHD types rated as useful by at least 50% of the surveyed HCPs qualified for the reporting. For epilepsy patients, heart rate, sleep quality, body movement^29,34^, breathing rate, mood and concentration^29^ were identified as useful. Sleep quality was also considered valuable for mental health and multiple sclerosis patients^29^. In geriatric care, HCPs rated heart rate, blood pressure, blood glucose, weight, body fat, body/skin temperature, physical activity, sedentariness, step count and electrodermal activity as useful^32^. In a study involving oncology care physicians, useful features for an oncology app would allow the tracking of side effects, quality of life, test results, and treatment satisfaction^25^. A few studies report on patient-reported outcome measures (PROMs), a subset of PGHD that is collected through standardised and validated questionnaires^10,11,21,24,25,32,33^. One study with mental healthcare professionals highlighted the value of combining PGHD from different sources, e.g., PROMs and app behavioural data, for a holistic view of a patient’s health status^21^. In contrast, GPs in another study valued PROMs collected through their own clinical system more valuable than other types of patient-collected data, such as wearable data^24^. For oncology patients, app-collected PROMs such as quality of life^25^ and therapy-related measures^10^ were considered valuable. One study specifically excluded PROMs from their investigation^11^. Table 3 shows a summary of all devices and respective PGHD types shared and/or discussed in the studies.

Despite the anticipated benefits of PGHD in patient care, HCP participants across the studies expressed mixed opinions about its nature, including both actively- (user input) and passively-sensed data. GPs of one study equated heart and sleep data from wearables to verbal symptom descriptions, not as concrete measures^24^. Conversely, others viewed PGHD as a more objective and potentially accurate source of medical information^20,26,35^, comparing it to “hard data”^28^ and considering it less prone to recall bias^35,37,39^. This digital data can also help avoid “doctor-pleasing” bias, where patients report what they think physicians want to hear^16,37^. Some HCPs appreciated the subjective nature of PGHD as a feature, acknowledging that it requires an understanding of how individual patients express themselves^39^.

For instance, a cardiologist in the same study described that managing atrial fibrillation often relies on patients’ subjective symptom experiences^39^. Additionally, a HCP in another study found that PGHD can provide a holistic view of a patient’s daily life, offering insights beyond what can be discussed in a single clinical visit and fostering greater empathy with patients^11^.

Even when PGHD did not directly impact care plans^37^ or was not seen as valuable evidence^24,35^, HCPs still found it a useful starting point for patient conversations and setting the consultation agenda^24,35,37^. The initiation of the PGHD tracking itself can help HCPs understand intrinsic patient behaviour and motivation^22,39^.

### Improving patient management and clinical workflows

In addition to its various benefits for patient care, HCPs across studies highlighted the potential of PGHD to enhance patient management and streamline clinical workflows. Figure 1b provides a summary of these findings.

In several studies, HCPs noted the utility of PGHD in preparing consultations. By tailoring appointments to specific patient issues identified in the PGHD, consultations could become more patient-centred and efficient^9,10,20,22,25,29,37,40^. For example, 77.8% of oncology care professionals in one study anticipated more efficient consultations with PGHD access^25^, while only 18% of HIV-care professionals in another study expected similar time savings^12^. In a study piloting a diabetes report, HCPs described a possible successful PGHD integration in clinical workflow by standardising and automating patient self-assessment and providing HCPs with a structured report, preferred directly in the EHR system before the consultation^37^. A study involving rheumatoid arthritis patients who tracked daily, weekly, and monthly symptoms in an app directly synced with the EHR found that accessing this data during consultations was considered useful by treating HCPs^9^. The longitudinal view of the patient’s health status provided by the app was seen as a potential time-saving alternative to standard disease history-taking during consultations.

HCPs in several studies identified the value of accessing PGHD between clinical visits. This data can help prioritise patients for follow-up visits and reduce unnecessary consultations^18,19,25,29,31,35^, freeing up crucial resources in an overburdened healthcare system. For example, HCPs considered post-discharge wound data tracked by at-risk-patients at home with their phone valuable for triage^18^. Additionally, PGHD can facilitate remote monitoring^8–11,16–20,25,28–30,37–40^, which is particularly useful for conditions like diabetes, where therapy adjustments can be made without in-person visits^19,30^. This approach helps manage increasing service demands and staff shortages effectively. Similarly to the diabetes case, a study on epilepsy, depression, and multiple sclerosis found that using patients’ devices to collect data at home and directly send it to the EHR system allowed for review between consultations if the system flagged reasons for concern^29^.

An important discussion point for many participants was the timing (when) and tool (how) for accessing PGHD. Many HCPs preferred access to the PGHD directly before a consultation^20,34,37^, while others favoured during the visit^19,21,26,34,35^ or in between appointments^11,16,19,25,26,28^. Regardless of timing, most HCPs who were open to using PGHD preferred it integrated into the electronic health records (EHR) to streamline their workflow^8–11,15–22,25,28,33,37,39,40^. Some suggested labelling PGHD separately from clinical data within EHRs^28,33^, though a minority preferred keeping raw PGHD out of the EHR^28^. Additionally, some HCPs were positive about accessing PGHD through patient portals^16,22,33,34,38^ or on the device during consultations^19,21,22,25,26,34,39^.

PGHD are often unfamiliar data types and structures for the HCPs, as they are collected with consumer health technologies that are designed for the general public, not a medical audience. Following from this, HCPs expressed the need to rearrange the data and allow tailored visualisation options to make the PGHD more comprehensive and actionable^11,16,18,21,22,26,39^.

Preferences ranged from summary reports with labels for highlights or out-of-range values^18,26,37,40^ to full data access^28^.

For remote access to PGHD, HCPs emphasised the need for clear protocols and responsibilities for dealing with incoming data and suggested building on existing workflow strategies^18,22,25,34,37,40^. Many called for dedicated nurses or care coordinators to pre-process PGHD before involving physicians^8,17,18,22,25,29,34,37,40^. Gerontologic care clinicians expressed a desire for decision support systems to pre-process data and issue alerts^32^. However, physicians in a study piloting a diabetes PGHD report preferred to receive the report directly^37^.

It is important to note that the report in this study was tailored to the specific information needs of the treating physicians. When oncology HCPs were surveyed about an alert feature in an app, 64.8% supported notifications for critical data entries^25^. Of those, 49% wanted an alarm for the treating physician within 24 to 48 hours, 40% preferred immediate alarms for the physician on duty, and 14% wanted an independent query system. Epilepsy care professionals were less supportive of real-time alarm systems, preferring to use PGHD for assessing seizure events before or during consultations rather than for real-time monitoring^34^.

### Barriers to using PGHD in clinical practice

**Fig. 2:**
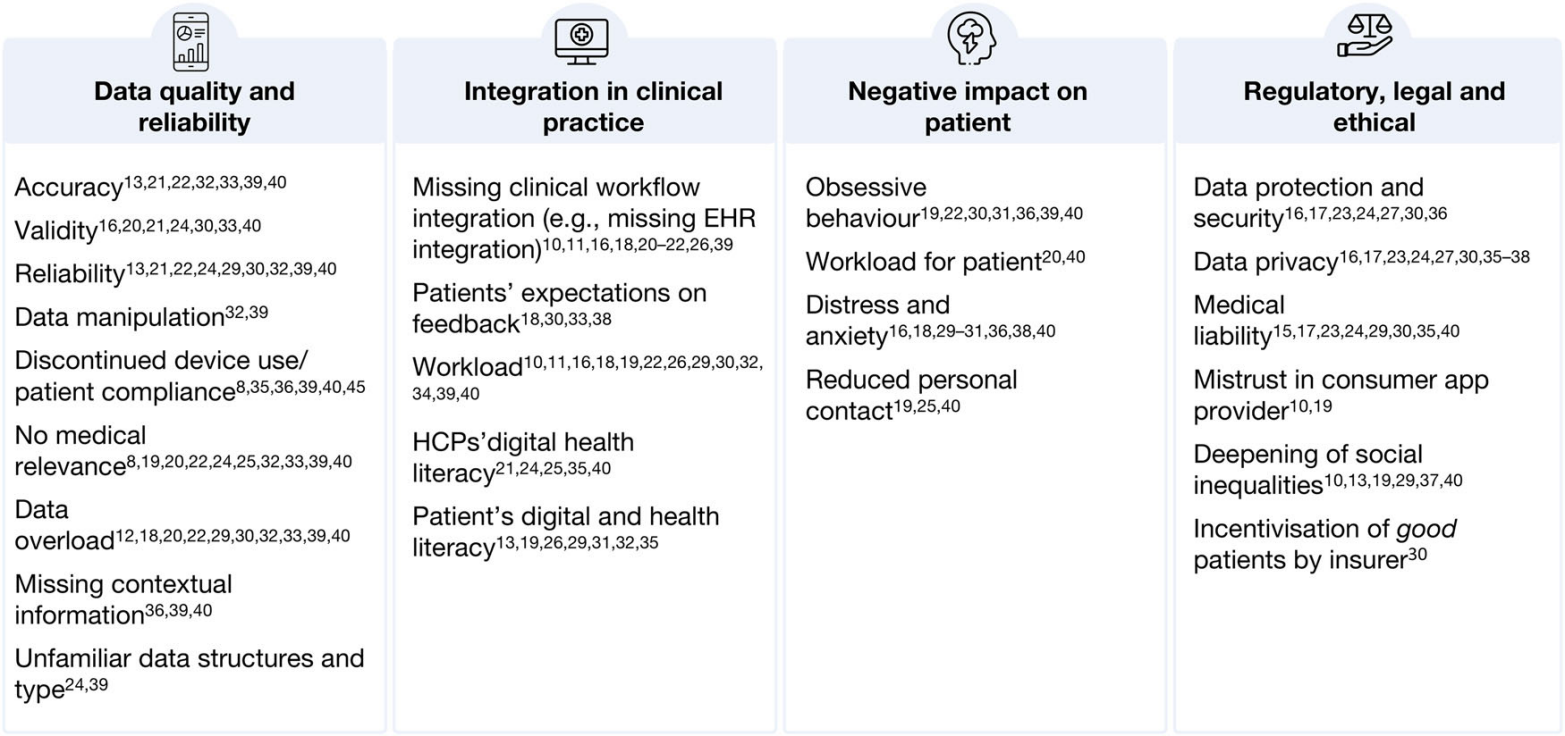
HCP concerns on integrating PGHD into care. Synthesised summary of HCPs’ concerns regarding the use of PGHD in clinical practice. (Author’s summary).

HCPs from various professional roles and medical specialities across studies, were enthusiastic about using PGHD to deliver better patient care, optimise patient management and streamline clinical workflows. However, the HCPs also reflected a variety of barriers and concerns when using this data. This shows a dual attitude of HCPs towards PGHD from health apps and wearables. Figure 2 summarises the barriers and concerns HCPs expressed in the studies included in this review.

A central barrier to HCPs was the quality and reliability of PGHD and the impact on data evaluation. Across studies, worries around data accuracy^13,21,22,32,33,39,40^, validity^16,20,21,24,30,33,40^, and reliability^13,21,22,24,29,30,32,39,40^ were found as a major concern, indicating an underlying mistrust in consumer technologies. On one hand, this is rooted in HCPs not knowing how the devices work and how they measure data. On the other hand, HCPs doubt patients’ ability to use the device correctly and trustworthy. Some HCPs feared that their patients could purposely try to manipulate the data to mislead physicians and force specific actions, e.g. a specific diagnosis or insurance premium^32,39^. Additionally, just like in classic therapies, digital approaches are facing big challenges in patient compliance. A non-compliant or discontinued use of the device could make the PGHD further unreliable, as some HCPs noted^13,29,32,33,38,40^. While access to more data is generally valued, HCPs in many studies are worried about data overload^12,18,20,22,29,30,32,33,39,40^. This could become particularly stressful for HCPs when patients gather data deemed to have no medical relevance^8,19,20,22,24,25,32,33,39,40^. Regarding passively sensed PGHD, HCPs expressed concerns about the missing contextual information, which makes it difficult to interpret the data^36,39,40^.

Another big concern theme was the integration of PGHD into current clinical practice. In several studies, HCPs noted that PGHD are not integrated into the current clinical workflow, e.g., missing interoperability with running information management systems in clinics^10,11,16,18,26,39^. It is time-consuming and burdensome for HCPs to assess PGHD in a separate software from their EHR system. Another barrier is the workload^10,11,16,18,19,22,26,30,32,34,39,40^ caused by the additional data. Processing and reviewing the data would require additional time and staff resources, especially in remote monitoring settings. Asynchronously sharing PGHD with HCPs brings the additional challenge of patients’ expectations on response time^18,30,33,38^ and HCPs noted the need for a clear alignment (and control over) when and how patients would receive a response to shared PGHD^11,18,38^.

Another important concern raised by HCPs in several studies that made the integration of PGHD in clinical routines difficult was the varying levels of digital health and data literacy among healthcare professionals, which can negatively impact how they interact with the tools and the data collected by patients^21,24,25,35,40^. On the other hand, patients lacking digital health literacy were also of concern, as this can result in wrong application usage or misinterpretation of data^13,19,26,31,32,34,35,40^.

While apps and wearables are often celebrated as empowering tools for patients, HCPs in several studies worried about their potential negative impact on patient health. HCP worried that PGHD tracking could lead to obsessive behaviour in some patients or exacerbate existing tendencies in others^19,22,30,31,36,39,40^. The tracking could become an additional work for patients who already deal with a severe condition^20,40^. Here, HCPs not only recognise their own extra work but also respect the workload on the patient site. A constant engagement with one’s own health status can also cause distress and anxiety^16,18,29–31,36,38,40^, for example, when data shows unfavourable behaviour of the patient or certain goals are not achieved. The increasing digitalisation of the healthcare and the patient-provider relationship was also negatively seen by some HCPs who worried about reduced personal contact of patients with their HCPs could have on them^19,25,40^.

Another set of concerns can be grouped as regulatory, legal and ethical concerns connected to using PGHD. An often cited concern by HCPs related to data privacy^11,12,18,19,22,25,30,31,34,40^, protection and security^11,12,18,19,22,25,29^ issues, such as third-party access and storage of PGHD from consumer mHealth devices. Some HCPs were generally worried by the idea of using tools from commercial companies, so the source of the app or device mattered to them^10,19^. HCPs in several studies were further concerned about medical liability, especially about their responsibility of reviewing large amounts of data or using the information for patient prioritisation^10,12,18,19,24,25,33,40^. Another important concern raised by HCPs in several studies is the fear of deepening social inequalities among patients with varying socio-economic levels^10,13,19,34,37,40^. Patients of lower socioeconomic status often have limited access to technology, including the internet and devices, and can lack the ability to use digital tools effectively. As a result, patients who would benefit the most can not participate or can participate less effectively. This could be further exacerbated in a scenario identified by HCPs in a Swiss study^30^. They worried that apps and wearables could be used by insurers to incentivise and reward “good patients” for achieving health goals while leaving other patients with higher needs for activation behind.

### HCPs recommendation to address PGHD challenges

To address concerns around PGHD quality and reliability, HCPs in several studies demanded evidence proof for apps and wearables to be used in clinical care^8,10,15,26,33^. Making this information available to PGHDs would help them to identify appropriate tools and boost trust to use them with their patients. Greater involvement of HCPs in developing apps and devices could further enhance clinical usefulness^13,30^ and allow for the integration of customised features like tailored tracking features or data displays^11,18,26,28,39^.

To integrate PGHD in clinical workflows, the majority of HCPs wished to access the PGHD directly through their EHR system or patient portal^15,17,18,20,22,25,28,32–34,37^. HCPs in one study, however, expressed the need to clearly label the source of this information to differentiate it from other forms of clinical data^33^. For remote monitoring settings, HCPs suggested a dedicated nurse or care coordinator who could preprocess data and alarm physicians accordingly^8,17,18,22,29,34^. This suggestion builds on existing clinical structures where nurses are often the first point of contact for the patients. Clear reimbursement plans were also recommended by HCPs in several to compensate the extra work created by guiding patients in PGHD collection and using the data^16,18,22,33^.

Educational programmes for both HCPs and patients were identified as necessary to improve digital literacy^15,26,29,30^. For HCPs, this would enhance their ability to recommend and interact with PGHD tools, including data interpretation. For patients, these programmes could help to ensure correct usage and accurate data collection.

HCPs called for a clear regulatory framework addressing regulatory and ethical concerns such as data privacy and data security measures as well as third-party access to data^19,30^. Additionally, HCPs called for financial support to help patients from lower socioeconomic backgrounds to access PGHD tools^31^.

### Evolving patient-provider roles in a changing system

Traditionally, the healthcare system has been dominated by paternalistic structures, with physicians being the primary decision-makers in patients’ care journeys. This is rooted in the belief that physicians possess superior medical knowledge and expertise, making them the most capable of judging what’s best for patients. However, in recent years, medicine has become gradually more participatory. This trend is supported by the emerging use of consumer health technologies that offer individuals unprecedented access to health information, health tracking features and personalised data insight reports. These advancements create opportunities for shared decision-making and a participatory approach to medicine, transforming the patient-provider relationship into a more equitable, empowered dynamic. HCPs in numerous studies in this review noted the potential of apps and wearables for collaboration on personal health goals and jointly deciding on PGHD to be integrated into care plans^11,16,18,19,22,28,33,35,39,40^. This approach effectively balances patients’ needs with clinical relevance while mitigating the risk of overburdening HCPs with excessive and unnecessary PGHD. However, acceptance of this approach varied depending on the clinical setting, highlighting the contrast between acute and paternalistic versus chronic and participatory medicine. For instance, physicians and cardiologists in two studies found limited utility of PGHD in acute or emergency care settings, where such data might impede timely care delivery^24,40^. Another study reported a lack of interest in PGHD from surgeons, whereas cardiologists found the data valuable and speculated that paternalistic structures may remain relevant in acute care settings but are outdated for long-term chronic disease management^39^. Given the increasing prevalence of chronic conditions and the subsequent rising demand for health services, engaging patients in proactive self-management will be crucial.

Some HCPs in chronic care^20,37^, acute, post-surgery care settings^18^ or not-specified care^22^ prefer to control which patients track what types of data to ensure medical relevance and integration into care plans, avoiding unnecessary workload^18,20,22,37^. They were holding on to traditional paternalistic thinking. Many HCPs also wanted only selected patients to share PGHD^11,18,20,35,37^. Suggested criteria included patients with memory issues^35,37^, poorly managed conditions^11,37^, patients at risk^18^, or with sufficient digital literacy^20^. HCPs emphasised the need to protect patients for whom an excessive focus on PGHD could be stressful^19,22,29,30,39^, particularly those predisposed to obsessive behaviour or mental disorders^19,22,39^. Some GPs expressed concerns about an emerging “entertainment medicine”, where healthy individuals who need it the least engage in excessive tracking and over-medicalisation of health^40^. Other GPs worried about “body estrangement” when measured data and bodily experiences do not match. Balancing these diverse perspectives is essential for the effective integration of PGHD into clinical practice^30^.

## Discussion

This review summarises HCPs’ and researchers’ perspectives on integrating PGHD from apps and wearables into clinical practice and research. HCPs and researchers across various professional roles and medical specialities have high expectations for PGHD, particularly for its ability to provide novel insights into patients’ daily lives. This enriches the understanding of diseases and has the potential to improve patient care^9–13,15–17,19–21,25,26,28–30,32,34,35,37–40^. Identifying personal health goals in collaboration with the patient can encourage patients to greater ownership, self-management and compliance^11,12,15–17,19–22,28–30,34,35,37–40^. This is particularly important for ageing populations with rising prevalences of chronic diseases. However, the scientific evidence on the effectiveness of health apps and wearables is still limited and further research is needed^41,42^.

Using PGHD for remote check-in and prioritisation of care can reallocate staff and time resources in overstrained healthcare systems and enhance patient management and clinical workflows^8–11,16–20,22,25,28–30,34–40^.

PGHD from consumer health technologies presents several challenges. HCPs had major concerns regarding data quality, validity and reliability^13,16,20–22,24,29,30,32,33,39,40^. These concerns are not only rooted in a mistrust of the technology itself but also in patients’ ability to use the tools correctly, especially if they lack necessary digital health literacy^13,29,32,33,39,40^. Unfamiliar data types^24,39^ and unclear medical evidence^8,16,26,30,33,39,40^ further complicate the integration of PGHD. Additionally, missing integration in EHR systems^10,11,16,18,20–22,26,39^, along with concerns about data privacy, security, and protection^11,12,18,19,22,25,29–31,34,40^, poses significant barriers. Researchers further highlighted the importance of multidisciplinary teams^14,27^ and adaptation of protocols when working with PGHD^16^.

The benefits of PGHD, such as continuous monitoring, personalised treatment, and patient empowerment, align with evolving regulatory and policy frameworks aimed at enhancing patient-centred and data-driven healthcare, like the European Health Data Space^4^ or Health Data Usage Act^43^ in Germany. Moreover, integrating the large, diverse datasets collected through health apps and wearables with existing health data sets (e.g., clinical data) can support data-intensive secondary use cases such as medical research and health AI development^44^. The concerns among HCPs about the quality and reliability of the data from apps and wearables^13,16,20–22,24,29,30,32,33,39,40^ highlight the need for regulatory standards for consumer health technologies when data is supposed to be used for patient care or research. Regulatory bodies must establish and enforce guidelines for data accuracy, validation, and interoperability to address concerns. For instance, Germany developed an approach for apps on prescription, enabling app manufacturers to qualify for the statuary health insurance reimbursement scheme if the app meets the specific criteria on evidence, interoperability and data privacy and security^45^. It remains to be seen how the EHDS can fulfil the high expectations of HCPs, researchers, industry players and related stakeholders on a safe and secure data exchange platform.

**Fig. 3:**
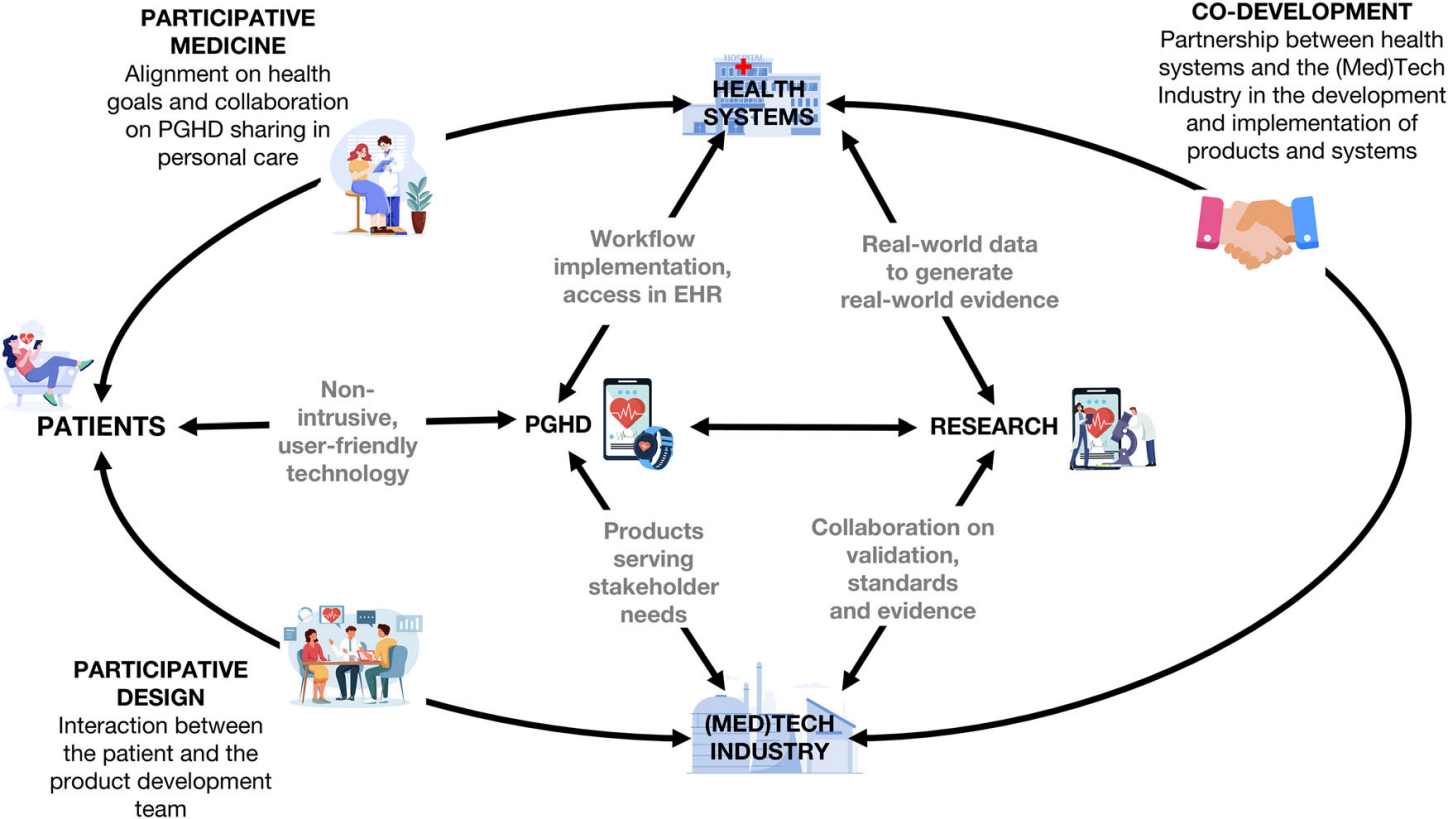
A collaborative framework for patient-generated health data (PGHD) in healthcare and innovation. (Author’s summary).

Apps and wearables on the consumer health technology market are primarily designed to meet the needs of their (paying) end users, typically individuals from the general public. However, integrating these tools into clinical care requires a collaborative effort involving app manufacturers, HCPs, health systems, patients, and researchers throughout the product development cycle. Such collaboration can facilitate the creation of tools that are user-friendly, scientifically validated, and clinically relevant^46^. Figure 3 illustrates this collaborative ecosystem to leverage the potential of PGHD. These practices are more common in the development of medical device apps - apps with a medical intended purpose, such as diagnosis or treatment - that are regulated by medical device regulations (e.g., FDA in the USA or MDR in the European Union)^47^. In contrast, wellness or lifestyle tools, such as fitness trackers or sleep monitors, are not designed for specific medical indications or with HCPs in mind but remain the most widely used. Making such data accessible to patients and HCPs will require innovative approaches, and as part of these, it is likely that artificial intelligence will be used in the processing and appropriate contextualising of information. For example, customised smart algorithms in EHR systems could be utilised to highlight to HCPS out-of-range data from individual or combinations of wearable devices, thereby enhancing accessibility^48^.

As identified by HCPs in several studies^10,13,19,30,34,37,40^, ethical and equity concerns must be addressed, as they hold significant societal implications. Digital transformation has the potential to create more equitable access to healthcare, alleviating current barriers for underserved populations. But without clear policies and social support systems, it can also reinforce existing social, economic, and health disadvantages, for example for the elderly or people with disabilities, subsequently deepening the digital divide^49^. A current initiative by the United States Food and Drug Administration FDA explores how healthcare at-home settings have to be designed to allow integrated digital care with a particular focus on equity^50^. For PGHD to be effectively integrated into healthcare, reimbursement schemes must cover the costs of devices and training, ensuring inclusive participation. Additionally, safeguards must be implemented to prevent data exploitation by commercial entities providing devices and apps.

Our review adds valuable insights into the perspectives and everyday experiences of PGHD from apps and wearables among HCPs and researchers, contributing to the growing body of evidence in this area. A recent review specifically focusing on HCPs’ real-world primary use cases of PGHD identified three motivational areas of HCPs to use PGHD: supporting patients in self-management, enhancing the patient-provider-relationship and supporting HCPs in their clinical roles^51^. The findings of our review on anticipated patient care benefits, patient management and clinical workflows align and expand on those findings while additionally reflecting in depth on a variety of concerns and barriers to PGHD usage, for example, fundamental data work challenges and worries of an over-technologized approach to medicine with reduced in-person contacts that could negatively impact the patient-provider relationship. Those findings are consistent with those in other literature^7^. Hence, at the core of PGHD and participatory medicine paradigm is the principle of collaboration, which requires compromises on patient and provider sites to leverage its greatest potential. Digital transformation often necessitates a fundamental reworking of processes and structures, not merely the addition of new tools to existing clinical workflows. As healthcare becomes more data-driven, roles and workflows will evolve. While some patients will become more participatory on their own, it will be particularly important for physicians to activate the less engaged but who would benefit the most from digital health interventions. It will be important to educate HCPs and patients on digital health literacy^13,15,19,21,24–26,29–32,35,40^. This topic not only has to be more integrated into the medical study curricula and continued training of physicians^52^, but also in the training of non-physician care professionals^53^. They are often the first point of contact for patients and are crucial in guiding them on the digital journey.

The strength of this review is that it considers studies of a broad range of care settings (clinic and outpatient care), healthcare provider and researcher professions and medical specialities. It was further not restricted to a specific disease indication or purpose of PGHD collection. Overall, it summarises a variety of opinions from healthcare providers and researchers that hold valuable insights into motivations to use this data as well as concerns around this usage.

This review has some limitations. First, there is a risk of missing studies during the search, despite our efforts to minimise this by searching multiple databases and refining search terms. We piloted search terms and used various synonyms to maximise the sensitivity of the search. There might be studies on data from apps and wearables that use less common words for describing PGHD or tracking devices and were missed by our search strings. Second, we limited our search to English and German original research articles. Third, this review did not select studies only involving HCPs with experiences in PGHD usage, which may have introduced complexity due to a heterogenic experienced population. However, the analysis of the included studies showed similar benefits and barriers themes across all studies and stakeholders, suggesting a limited impact of this factor. As more apps and wearables are implemented in clinical care, focusing on assessing real-world experiences and needs might reveal additional insights.

Several areas require further investigation to optimise the use of PGHD. Longitudinal studies could investigate the impact on patient outcomes, cost-effectiveness and workflow efficiencies. Research could also focus on developing standardised protocols for data collection, validation and PGHD integration as well as the integration with clinical data. Comparing HCP perspectives before and after the COVID-19 pandemic could offer insights into shifts in attitudes and clinical workflow impacts.

PGHD from apps and wearables hold significant promise for advancing patient care, optimising clinical workflows and supporting medical research. Addressing the challenges identified by HCPs will be crucial for its successful integration into clinical practice. By focusing on regulatory needs, evidence generation, workflow integration, as well as HCP and patient education, stakeholders can work towards harnessing the full potential of PGHD while ensuring its safe and equitable use.

## Methods

This review was registered with OSF (registration 10.17605/OSF.IO/PCZAU) and adhered to the Preferred Items for Systematic Reviews and Meta-Analyses statement (PRISMA)^54^. The PRISMA checklist can be found in Supplementary Information Table 1. Updates are accessible in the registry. Additional deviations to the protocol are listed in Supplementary Information Note 1.

### Search strategy

The literature search was conducted between April 11 to April 24, 2024. We searched the databases Pubmed, Embase and Google Scholar with search strings considering variations of the terms “healthcare professionals”, “researchers”, “health apps”, “sharing” and “data” for publications published between January 2013 and April 2023. Complete search strings are shown in Supplementary Information Note 2. To supplement database searches, the reference lists of included publications were searched (backward citation tracking) and articles that referenced the identified publication (forward citation tracking), as well as an additional hand search with Google.

### Eligibility criteria

Eligibility of records was assessed using the PICO framework to define inclusion and exclusion criteria based on the four key components: Population, Intervention, Comparison, and Outcome, and the additional criteria of Type of Study, Language of Publication, and Publication Date (Table 5).

**Table 5:** PICO framework inclusion and exclusion criteria (HCP healthcare professional; IT information technology; PGHD patient-generated health data)

Criteria	Inclusion	Exclusion
Population	HCPs defined as currently employed or practicing in providing care for patients, including physicians, nurses and therapists Researchers defined as individuals conducting research in healthcare, e.g., biomedical researcher, informatic researcher, social science researcher	Studies focusing solely on patients or consumers of health apps and wearables Perspectives of non-healthcare-related professionals (e.g., IT professionals, healthcare administrative staff)
Intervention	Primary or secondary use of at-home collected, patient-generated and shared health data from apps and wearables	Studies on medical data that is not collected by patients outside of the clinic Studies on non-health-related data from apps and wearables
Comparison	Not applicable	Not applicable
Outcome	Studies focusing on perspectives, attitudes, beliefs, or experiences related to: a) Primary use of PGHD: Use of data for immediate patient care, diagnosis, treatment, and monitoring. b) Secondary use of PGHD: Use of data for research, quality improvement, public health, and policy-making.	Studies not addressing the primary or secondary use of PGHD Research on methods and concepts for technology development, assessments and implementation without considering the perspectives of HCPs and researchers Regulatory assessments
Types of studies	Qualitative, quantitative and mixed-method studies	Reviews, books or non-empirical research (e.g. systematic reviews, opinion pieces, theoretical papers without primary data)
Further criteria	Language of publication English or German Studies published between January 2013 – April 2023 Studies available in full-text format	Publication not available in English or German and not in full-text format

### Study selection and data extraction

After the search, all retrieved bibliographic data were imported to Rayyan^55^ and duplicates were removed. A test screening was conducted with 20 publications (8% of the total number of articles) to refine the inclusion criteria and ensure consistency among reviewers in the study selection. First, authors SB and OS independently screened titles and abstracts to find publications that meet the inclusion criteria. Second, SB and OS independently assessed full texts to identify eligible studies for inclusion in the review. SB and OS used a Google Survey Form and Excel to extract data from all included studies. Extracted data consisted of metadata of the article and information related to the research questions, such as study type, mHealth technology investigated, use cases, benefits and concerns. All extracted data can be found in Supplementary Information Table 3. Table 2 shows a summarised version of the extracted data.

### Quality assessment

SB and OS independently assessed the quality of the included studies using the Mixed Methods Appraisal Tool (MMAT) Version 2018 (Table 4)^56^ Disagreements at any stage were resolved by discussion with SG.

### Data synthesis and analysis

A data-based convergent synthesis design^57^ was used to analyse all included studies following a thematic synthesis approach. In this approach, all quantitative data (numerical results from quantitative and mixed methods studies) is transformed into codes and analysed with the qualitative data (qualitative results from qualitative and mixed methods studies). The thematic synthesis was performed based on the method by Thomas and Harden which includes line-by-line coding, grouping codes in descriptive themes and generating analytical themes^58^. The initial set of codes for the analytical themes was developed during the full-text screening. SB coded all studies using the software MaxQDA (VERBI Software GmbH). The data analysis followed an iterative approach, adding new codes every time a new theme was identified. After coding all articles, SB assessed the coding of all articles again to ensure consistency. Finally, analytical themes were identified and discussed among SB, OS and SG.

## Supplementary information

Supplementary information

## Data Availability

All data generated and analysed during this study are included in the article and its Supplementary Information.

## Abbreviations

AF: Atrial fibrillation
AUS: Australia
BE: Belgium
CA: Canada
CGM: Continuous Glucose Monitoring
CH: Switzerland
DE: Germany
DK: Denmark
EHDS: European Health Data Space
EHR: Electronic health record
FR: France
GP: General practitioner
HCP: Healthcare professional
HIV: Human immunodeficiency virus
IRE: Ireland
MS: Multiple sclerosis
NL: Netherlands
PGHD: Patient-generated health data
PROMs: Patient-reported outcome measures
RA: Rheumatoid arthritis
UK: UK United Kingdom
USA: United States of America
VA: U.S. Department of Veterans Affairs
