# Supplementary material for: Attitudes of healthcare professionals and researchers toward wearable and app derived patient generated health data
Source: NPJ Digit Med. 2025 Mar 30;8:186. doi: 10.1038/s41746-025-01568-4 (PMC11955519; doi:10.1038/s41746-025-01568-4)
Supplement: Supplementary file 1 — Supplementary information [file 41746_2025_1568_MOESM1_ESM.pdf]

Supplementary Information

Content

Supplementary Information Table 1: Prisma Checklist
Supplementary Information Note 1: Protocol deviations
Supplementary Information Note 2: Search strings
Supplementary Information Table 2: Table of exclusion reasons for full texts reviewed
Supplementary Information Note 3: Researchers’ perspective
Supplementary Information Table 3: Data extraction

Supplementary Information Table 1: Prisma Checklist

PRISMA 2020 Checklist

Section and Topic	Item #	Checklist item	Location where item is reported
**TITLE**			
Title	1	Identify the report as a systematic review.	1
**ABSTRACT**			
Abstract	2	See the PRISMA 2020 for Abstracts checklist.	n.a.
**INTRODUCTION**			
Rationale	3	Describe the rationale for the review in the context of existing knowledge.	2
Objectives	4	Provide an explicit statement of the objective(s) or question(s) the review addresses.	2
**METHODS**			
Eligibility criteria	5	Specify the inclusion and exclusion criteria for the review and how studies were grouped for the syntheses.	13. Suppl. Information Section 4
Information sources	6	Specify all databases, registers, websites, organisations, reference lists and other sources searched or consulted to identify studies. Specify the date when each source was last searched or consulted.	13
Search strategy	7	Present the full search strategies for all databases, registers and websites, including any filters and limits used.	13, Suppl. Information Section 3
Selection process	8	Specify the methods used to decide whether a study met the inclusion criteria of the review, including how many reviewers screened each record and each report retrieved, whether they worked independently, and if applicable, details of automation tools used in the process.	13
Data collection process	9	Specify the methods used to collect data from reports, including how many reviewers collected data from each report, whether they worked independently, any processes for obtaining or confirming data from study investigators, and if applicable, details of automation tools used in the process.	13
Data items	10a	List and define all outcomes for which data were sought. Specify whether all results that were compatible with each outcome domain in each study were sought (e.g. for all measures, time points, analyses), and if not, the methods used to decide which results to collect.	13
	10b	List and define all other variables for which data were sought (e.g. participant and intervention characteristics, funding sources). Describe any assumptions made about any missing or unclear information.	13
Study risk of bias assessment	11	Specify the methods used to assess risk of bias in the included studies, including details of the tool(s) used, how many reviewers assessed each study and whether they worked independently, and if applicable, details of automation tools used in the process.	13
Effect measures	12	Specify for each outcome the effect measure(s) (e.g. risk ratio, mean difference) used in the synthesis or presentation of results.	n.a.
Synthesis	13a	Describe the processes used to decide which studies were eligible for each synthesis (e.g. tabulating the study intervention characteristics and	13-14

Section and Topic	Item #	Checklist item	Location where item is reported
methods		comparing against the planned groups for each synthesis (item #5)).	
	13b	Describe any methods required to prepare the data for presentation or synthesis, such as handling of missing summary statistics, or data conversions.	n.a.
	13c	Describe any methods used to tabulate or visually display results of individual studies and syntheses.	n.a.
	13d	Describe any methods used to synthesize results and provide a rationale for the choice(s). If meta-analysis was performed, describe the model(s), method(s) to identify the presence and extent of statistical heterogeneity, and software package(s) used.	13-14
	13e	Describe any methods used to explore possible causes of heterogeneity among study results (e.g. subgroup analysis, meta-regression).	n.a.
	13f	Describe any sensitivity analyses conducted to assess robustness of the synthesized results.	n.a.
Reporting bias assessment	14	Describe any methods used to assess risk of bias due to missing results in a synthesis (arising from reporting biases).	n.a.
Certainty assessment	15	Describe any methods used to assess certainty (or confidence) in the body of evidence for an outcome.	n.a.
**RESULTS**			
Study selection	16a	Describe the results of the search and selection process, from the number of records identified in the search to the number of studies included in the review, ideally using a flow diagram.	Table 1
	16b	Cite studies that might appear to meet the inclusion criteria, but which were excluded, and explain why they were excluded.	Table 1
Study characteristics	17	Cite each included study and present its characteristics.	2-9, Table 2, Suppl. Information Section 6
Risk of bias in studies	18	Present assessments of risk of bias for each included study.	3-4, Table 4
Results of individual studies	19	For all outcomes, present, for each study: (a) summary statistics for each group (where appropriate) and (b) an effect estimate and its precision (e.g. confidence/credible interval), ideally using structured tables or plots.	2-9, Suppl. Information Section 5
Results of syntheses	20a	For each synthesis, briefly summarise the characteristics and risk of bias among contributing studies.	n.a.
	20b	Present results of all statistical syntheses conducted. If meta-analysis was done, present for each the summary estimate and its precision (e.g. confidence/credible interval) and measures of statistical heterogeneity. If comparing groups, describe the direction of the effect.	n.a.
	20c	Present results of all investigations of possible causes of heterogeneity among study results.	n.a.
	20d	Present results of all sensitivity analyses conducted to assess the robustness of the synthesized results.	n.a.
Reporting biases	21	Present assessments of risk of bias due to missing results (arising from reporting biases) for each synthesis assessed.	n.a.
Certainty of	22	Present assessments of certainty (or confidence) in the body of evidence for each outcome assessed.	n.a.

Section and Topic	Item #	Checklist item	Location where item is reported
evidence			
**DISCUSSION**			
Discussion	23a	Provide a general interpretation of the results in the context of other evidence.	10-12
	23b	Discuss any limitations of the evidence included in the review.	12
	23c	Discuss any limitations of the review processes used.	12
	23d	Discuss implications of the results for practice, policy, and future research.	10-12
**OTHER INFORMATION**			
Registration and protocol	24a	Provide registration information for the review, including register name and registration number, or state that the review was not registered.	12
	24b	Indicate where the review protocol can be accessed, or state that a protocol was not prepared.	12
	24c	Describe and explain any amendments to information provided at registration or in the protocol.	12, Suppl. Information Section 2
Support	25	Describe sources of financial or non-financial support for the review, and the role of the funders or sponsors in the review.	14
Competing interests	26	Declare any competing interests of review authors.	14
Availability of data, code and other materials	27	Report which of the following are publicly available and where they can be found: template data collection forms; data extracted from included studies; data used for all analyses; analytic code; any other materials used in the review.	Suppl. Information

From: Page MJ, McKenzie JE, Bossuyt PM, Boutron I, Hoffmann TC, Mulrow CD, et al. The PRISMA 2020 statement: an updated guideline for reporting systematic reviews. BMJ 2021;372:n71. doi: 10.1136/bmj.n71

Supplementary Information Note 1: Protocol deviations

The protocol was registered on April 4, 2023. An update was issued August 5,2024 with the following changes:

1. Change of the study design to a mixed methods systematic review which is more suitable to answer our research question than the initially planned scoping review.
2. Narrowing the scope of the study from initially three research questions about the perspectives of citizens/patients, HCPs and other stakeholders towards primary and secondary use of PGHD from apps and wearables on two research questions investigating the perspectives of HCPs and researchers. The perspectives of citizens will be summarised in a separate publication.

Supplementary Information Note 2: Search strings

PubMed and Embase

1. (Doctors [Title/Abstract] OR Physicians[Title/Abstract] OR "Healthcare providers") AND ("health apps" OR ("wellness apps"[Title/Abstract] OR "wellness applications"[Title/Abstract]) OR "medical apps"[Title/Abstract] OR "wearable electronic devices" OR DIGA OR ("mobile applications"[Title/Abstract] OR "mobile health apps"[Title/Abstract] OR "mobile health applications") OR ("digital health apps"[Title/Abstract] OR "digital health applications")) AND (sharing OR transfer OR access OR gather OR collect) AND (("health data"[Title/Abstract] OR "healthcare data"[Title/Abstract] OR "health care data"[Title/Abstract]) OR "wellness data"[Title/Abstract] OR "wearable data"[Title/Abstract] OR "fitness data"[Title/Abstract] OR "lifestyle data"[Title/Abstract])

2. ("Stakeholders"[Title/Abstract] OR "Researchers"[Title/Abstract] OR "Regulators"[Title/Abstract] OR "policymakers ") AND ("health apps" OR ("wellness apps"[Title/Abstract] OR "wellness applications"[Title/Abstract]) OR "medical apps"[Title/Abstract] OR "wearable electronic devices" OR DIGA OR ("mobile applications"[Title/Abstract] OR "mobile health apps"[Title/Abstract] OR "mobile health applications") OR ("digital health apps"[Title/Abstract] OR "digital health applications")) AND (sharing OR transfer OR access OR gather OR collect) AND (("health data"[Title/Abstract] OR "healthcare data"[Title/Abstract] OR "health care data"[Title/Abstract]) OR "wellness data"[Title/Abstract] OR "wearable data"[Title/Abstract] OR "fitness data"[Title/Abstract] OR "lifestyle data"[Title/Abstract])

Google Scholar

1. (Doctors|physicians|healthcare providers) (“health apps”|“medical apps”|“wellness apps”|DIGA|“digital health apps”|“mhealth apps”|“mobile health apps”) (sharing|transfer|access|gather|collect) (“health data”| wellness data ”|“wearable data”|“fitness data”|“lifestyle data)

2. (Stakeholders|researchers|regulators|policymakers) (“health apps”|“medical apps”|“wellness apps”|DIGA|“digital health apps”|“mhealth apps”|“mobile health apps”) (sharing|transfer|access|gather|collect) (“health data”| wellness data ”|“wearable data”| “fitness data”|“lifestyle data)

Supplementary Information Table 2: Table of exclusion reasons for full texts reviewed

Authors (year)	Title	Journal	DOI	Exclusion reason
Byambasuren et al. (2020)	Barriers to and facilitators of the prescription of mHealth apps in Australian general practice: qualitative study	JMIR Mhealth Uhealth	10.2196/17447	Wrong outcome<br>Study examines barriers and facilitators for GPs to prescribing mHealth apps and patients using them but didn’t assess their perspectives on PGHD for primary and/or secondary use
Dahlhausen et al. (2021)	Physicians' attitudes toward prescribable mHealth apps and implications for adoption in Germany: mixed methods study	JMIR Mhealth Uhealth	10.2196/33012	Wrong outcome<br>Study investigates physicians and psychotherapist attitudes toward prescribing mHealth apps but didn’t assess their perspectives on PGHD for primary and/or secondary use
Hofer & Haluza (2019)	Are Austrian practitioners ready to use medical apps? Results of a validation study	BMC Medical Informatics and Decision Making	10.1186/s12911-019-0811-2	Wrong outcome<br>Study investigated physicians’ health app usage and related concerns but didn’t assess their perspectives on PGHD for primary and/or secondary use
Jezrawi et al. (2022)	Patient and physician perspectives on the use and outcome measures of mHealth apps: Exploratory survey and focus group study	Digital Health	10.1177/20552076221102773	Wrong outcome<br>Study examined factors influencing HCPs to recommend and patients to use health apps but didn’t assess HCPs perspectives on PGHD for primary and/or secondary use
Mueller (2020)	Exploring family nurse practitioners' practices in recommending mHealth apps to patients	CIN: Computers, Informatics, Nursing	10.1097/CIN.0000000000000580	Wrong outcome<br>Study investigated app prescription and usage of family nurse practitioner but didn’t assess their perspectives on PGHD for primary and/or secondary use

Murali-Ganesh et al. (2018)	From smartphone to electronic health record (EHR): An innovative implementation of patient-reported outcomes and patient-generated health data in routine cancer care	Asia-Pacific Journal of Clinical Oncology	10.1111/ajco.13089	Full text not available
Nogueira-Leite & Cruz-Correia (2023)	Attitudes of physicians and individuals toward digital mental health tools: Protocol for a web-based survey research project.	JMIR research protocols	10.2196/41040	Wrong outcome<br>Study assessed HCPs perspectives on health apps in general, but not on the generated data for primary or secondary use
Sezgin et al. (2017)	Investigation of physicians' awareness and use of mHealth apps: a mixed method study	Health Policy and Technology	10.1016/j.hlpt.2017.07.007	Wrong outcome<br>Study investigated HCPs acceptance and usage of health apps, but didn't assess their perspectives on PGHD for primary and/or secondary use
Sprenger et al. (2017)	Health professionals' perspective on the promotion of e-mental health apps in the context of maternal depression	PLOS One	10.1371/journal.pone.0180867	Wrong outcome<br>Study focused on HCPs intentions to use and recommend health apps but didn't assess their perspectives on PGHD for primary and/or secondary use
Vecchia et al. (2022)	Willingness of French general practitioners to prescribe mhealth apps and devices: quantitative study	JMIR Mhealth Uhealth	10.2196/28372	Wrong outcome<br>Study investigates factors influencing willingness of GPs to prescribe health apps but didn't assess their perspectives on PGHD for primary and/or secondary use
Zhang & Koch (2015)	Mobile health apps in Sweden: what do physicians recommend?	IOS Press Ebooks - Volume 210: Digital Healthcare Empowering Europeans	10.3233/978-1-61499-512-8-793	Wrong outcome<br>Study focused on physician attitudes toward recommending health apps but didn't assess their perspectives on PGHD for primary and/or secondary use

Supplementary Information Note 3: Researchers' perspective

Three studies interviewed researchers about their experiences with PGHD. Participants came from diverse fields, including medical, social, life, and computational science (Table 2). Participants across all studies had high expectations for integrating PGHD in clinical care and research, providing new insights into patients' daily lives.[14,23,27] Researchers in gait and physical activity noted a paradigm shift where at-home sensor-collected data offer a new understanding of the disease, for example, by revealing new symptoms, and HCPs now consider available consumer technology for treatment plans.[14] For research, the key is integrating data from multiple sources, clinical data and non-clinical PGHD, to enrich understanding.[14,23,27] Further, researchers note that PGHD usage requires learning from trial and error with new devices and compromising on existing protocols to balance clinical and practical needs.[14] First, selecting relevant PGHD to answer clinical and research questions while avoiding unnecessary data or data overload is crucial.[14] In the same study, some researchers noted that the technology is still maturing and may not yet support clinical decision-making.[14] Second, choosing user-friendly, non-intrusive devices with a high compliance rate might mean sacrificing desired data.[14] This importance was also echoed by HCPs in primary care.[28,34] Participants stressed the importance of properly onboarding staff and patients to avoid complications and data issues.[14] Providing feedback to patients during trials was identified as essential to increase patient compliance and minimise dropout rates.[14]

Several challenges tempered participants' enthusiasm for PGHD. Researchers identified data quality as a major issue, as commercial devices often provide summary data rather than the raw data needed for research.[14] Even when raw data is available, problems such as proprietary algorithms, lack of standardisation, and unclear validation remain.[14,27] The lack of standards complicates comparing results across different studies.[14] Researchers also mentioned PGHD collected in everyday life is harder to interpret and contextualise than data from controlled lab environments.[27] Another challenge is related to staff and patients. Participants emphasised the importance of training for everybody involved to know their competencies, even if the collected data seemed to be "easy".[14] Multidisciplinary teams with engineers, computer scientists, HCPs and others were identified as key to successful PGHD usage.[14,27] While most researchers reported enthusiastic patients,[14] some also mentioned patient reluctance to record detailed information due to a fear of data misuse,[27] highlighting the importance of clear data governance and communication strategies. Additionally, a collaboration with a private device manufacturer poses a challenge. Researchers reported concerns about intellectual property, data licensing, legal arrangements, consent, legal frameworks and collaboration processes.[23] A researcher described a mismatch between expectations and current practice, where using PGHD in clinical trials slows down processes and increases workload.[14] In their view, the positive impact on costs, operational improvements, or quality of endpoints remains to be proven.[14]

Supplementary Information Table 3: Data extraction

Author (year)	Country	Objective	Journal	Study type	Medical professions (number of participants)	Other participants (excluded from analysis)	Recruitment	Data collection	Data analysis	Disease indication focus	Study part of clinical research program	mHealth technology	Type of PGHD collected or discussed	Provider access to PGHD	Main findings
Abdolkhani et al. (2019)[8]	AUS	To identify challenges regarding PGHD quality and potential solutions from the perspective of consumer, healthcare provider and app manufacturer	JMIR Mhealth UHealth	Qualitative	Clinicians (5)	Health consumers and advocates (8), health information professionals (3),<br>wearable and data integration company representatives (2)	Open call and a web-based registration form distributed via internal and public news and media channels and professional organizations for digital health in Australia; personal invitations and snowball sampling	Workshop with group discussions (in person)	Thematic analysis	Not specified	No	Wearables (medical grade and consumer technology)	CGM and fitness tracker data as example	Integration in EHR desired	HCPs identified various challenges regarding PGHD quality that need to be addressed to ensure its use. They view collaboration with patients on PGHD tracking and use (remote monitoring) as a partnership in which they (HCPs) should be the trusted first point of decision-making for patient care. Manufacturers of these tools should aim to collect clinical evidence and design tools aligned with the standards of healthcare settings.
Adler-Milstein & Nong (2019)[33]	USA	To characterize provider-led PGHD approaches, assess their alignment with patient preferences, and identify challenges to scaling and impact	Journal of the American Medical Informatics Association	Qualitative	Representatives of health systems (6)	Patients (10), EHR vendors (6)	Selective selection of health systems based on PGHD experience through the American Hospital Association's IT Supplement survey; EHR vendors recruited based on health system interviews; patient recruitment through the University of Michigan's Health Research platform based on inclusion criteria	Interview (phone)	Content analysis	Not specified	No	Various mHealth technologies, including patient's own devices, in-clinic tablets, and health system-provided devices	Health history, validated quenstionnaires and surveys (incl. PROMs), biometric data (e.g. blood pressure, weight, exercise, nutrition)	Different approaches to PGHD access within health systems, PGHD type depending, e.g., integration in EHR, patient portals or separate dashboards	Health system representatives identified three PGHD categories currently pursued: health history, validated questionnaires/surveys, and biometrics/activity (e.g., blood pressure). They also identified barriers to PGHD adoption: data value and quality, lack of reimbursement, and lack of workflow integration.
Andrews et al. (2022)[29]	UK	To explore the views of healthcare providers on remote monitoring through health apps and wearables for epilepsy, multiple sclerosis and depression	BMC Medical Informatics and Decision Making	Quantitative	HCPs (1006):<br>Doctor (excl. GPs) (138), GPs (118), research/healthcare science (24), management (40), nursing (268), pharmacy (15), psychological professions (157), student (10), wider healthcare team (76), not clear (48)	No	Advertising of study through East Midlands Clinical Research Network and RADAR-CNS research consortium, social media accounts, consortium website and consortium newsletter	Survey (online)	Frequencies, percentages and chi-squared analysis for quantitative results and content analysis for free text entry	Epilepsy, multiple sclerosis (MS) and depression	No	Health apps and wearables for remote measuring/monitoring	Disease-related actively and passively collected PGHD like body movement, heart rate, sleep and others	PGHD access during consultation via patients' device, automatic transfer anytime via a secure portal, remote access at patients' discretion, through a system with alarm feature	HCPs were positive about using PGHD for remote monitoring. Various PGHD types were considered helpful depending on the indication. PGHD should be accessible to the care teams before, during, and after consultations. Concerns were workload, increasing patient anxiety, and reinforcing health disparities.
Austin et al. (2020)[9]	UK	To evaluate a rheumatoid arthritis (RA) PGHD monitoring system for acceptability and feasibility (within REMORA study setting)	Rheumatology	Qualitative	Clinicians (2)	Patients (20)	Recruitment of patients and clinicians via rheumatology department (Salford Royal NHS)	Interview (not specified)	Thematic analysis (grounded theory approach)	Rheumatoid athritis	Remote Monitoring of rheumatoid arthritis (REMORA) study	REMORA smartphone app for patients with an EHR/research database integration	RA symptoms, daily disease impact	Graphical summaries of longitudinal data in research data base and EHR, accessed during consultation	Clinicians appreciated that the collected PGHD provided a bigger picture, identifying real-time changes in disease activity and capturing symptoms that would otherwise have been missed. PGHD can be used to set an agenda for a consultation and to support patients' memories.
Berkowitz et al. (2017)[10]	USA	To investigate healthcare provider perspectives on the opportunities and barriers for mHealth app use in oncology care	JCO Clinical Cancer Informatics	Qualitative	Oncology care professionals (15): Physicians (8), advanced practice providers, e.g. nurse practitioners, physician assistants (3), supportive service providers, e.g. social workers, nursing support staff (4)	No	Purposive sampling of oncology providers affiliated with Duke University Health System	Interview (in person or via phone)	Thematic analysis	Cancer	No	Health apps for cancer care	Examples included PROMs related to therapies	Integration in EHR desired	HCPs report limited exposure to oncology apps in patient care but were generally open to using them. Expected benefits are health promotion, symptom tracking and patient engagement. Perceived barriers are access to technology, responsibility, workflow disruption and the source of the app itself.
Bietz et al. (2016)[23]	USA	To understand experiences, utility and barriers to using personal health data in research from the perspectives of early adopters, researchers, and companies with experience in PGHD	Journal of the American Medical Informatics Association	Mixed methods	Researchers (134): Health Science, social science, life science, engineering and technology, arts and humanities	Patients (465), industry stakeholders (15)	Targeted convenience sampling of individuals and researchers with PGHD experience through postings on self-tracking related websites, relevant press releases and social media channels; after participating in survey, participants could opt-in for follow-up interviews; selection of companies of the PGHD economy not further specified	Survey (online), interview (in person or via phone)	Thematic analysis (grounded theory approach) for interviews; descriptive analysis for survey	Not specified	No	Health apps and wearables (consumer technology)	Various types, including vital signs, stress levels, mood and physical activity	Issues with data access from commercial companies discussed	Most researchers (89%) perceive PGHD as useful, especially vital signs, stress levels, and mood. Almost all researchers believe PGHD can answer questions traditional clinical data cannot. However, there are barriers to using PGHD, including data quality and validity, IP concerns, licensing, collaboration with companies, and others.
Bruno et al. (2018)[34]	UK	To investigate the perspectives of people with epilepsy, caregivers, and healthcare professionals on the current use of and willingness to use digital technology and wearables for seizure monitoring	Epilepsy & Behavior	Quantitative	HCPs (22): Neurologists (10), psychiatrist (1), epilepsy nurses (7), service managers (2), disability nurse (1), medical student (1)	Patients (52), caregivers (13)	Recruitment via weblink through various relevant channels, including two NHS trusts, epilepsy charities and posts on research network project website	Survey (online)	Descriptive and correlation analysis	Epilepsy	No	Health apps and wearables for remote monitoring	Epilepsy-related information through sensors, including seizure detection, sweat, and voice quality; less favoured were mood, concentration, attention and memory	On device during consultation or integration in patient portal	HCPs see the usefulness of digital tools for patient management (68.2%), especially for specific decision points (e.g., treatment adjustments); however, 40% state that this information is never or rarely used. Half of HCPs are concerned about increased workload due to data reviewing and suggest a nurse as an appropriate team member to deal with PGHD.
Cohen et al. (2016)[11]	USA	To examine the experiences of HCPs in outpatient clinics using PGHD in care as part of a national research initiative (Project HealthDesign program)	JMIR Human Factors	Qualitative	HCP (25): Physicians, nurses, health coaches (12);<br>study team members (13)	No	Participants were recruited through the five studies within the Project HealthDesign research project	Interview (in person or video conference platform)	Immersion-crystallisation approach	Chronic conditions (asthma, cognitive decline, overweight, Crohn's disease)	Project HealthDesign program	Consumer mHealth technologies	Various PGHD were collected: medication usage, peak flow, cognitive abilities, food intake, activtiy, weight, mood, and symptoms among others	Different PGHD access: Web-based dashboards or platforms as part of the respective research programs; if used in routine care, integration in EHR is desired	HCPs see benefits in PGHD access in three main areas: deeper insights into patients, more accurate patient information, and insights between clinical visits. PGHD implementation requires adaptation in clinical workflows and patient-provider alignment on communication expectations (when used in remote monitoring settings).
Gabriels & Moerenhout (2018)[40]	BE	To analyse how physicians evaluate classic and digital self-tracking in everyday clinical practice and to explore the impact of digital self-tracking on self-care and professional healthcare	JMIR	Qualitative	Physicians (12): GPs (7), cardiologists (5)	No	Purposive, convenience-based sampling	Interview (in person)	Thematic analysis	Not specified	No	Health apps and wearables (consumer technology)	Not specified	Integration in EHR desired	GPs and cardiologists had little experience with PGHD from consumer technologies, and it's not yet an integrated part of their clinical practice. The interpretation of this data, data overload, and the potential of an emerging "entertainment medicine" were concerns.

Author (year)	Country	Objective	Journal	Study type	Medical professions (number of participants)	Other participants (excluded from analysis)	Recruitment	Data collection	Data analysis	Disease indication focus	Study part of clinical research program	mHealth technology	Type of PGHD collected or discussed	Provider access to PGHD	Main findings
Haase et al. (2023)[24]	DK	To investigate GPs engagement with patient-provided data from new technologies	Social Studies of Science - Sage Journals	Qualitative	GPs (23)	No	Purposive sampling of GPs in various clinical settings and geographical diversity	Interview (in person)	Thematic analysis and Science and Technology Studies framework	Not specified	No	Wearables (consumer technology), online symptom checker (commercial company or health system provided)	Heart and sleep data from wearables, online symptom and health checker were compared to physician-initiated digital data (official lab test results and GP PRO system)	During consultation on device and often only verbal description	Only a few patients bring PGHD to a consultation (sleep data, heart rate data or symptom checker). Those PGHD are not analysed as measurements but more as another symptom description from the patient. Patient-initiated data generation was considered less relevant in comparison to healthcare system generated data - even if the underlying test is the same.
Huh et al. (2013)[35]	USA	To understand different healthcare professionals' and patients' perspectives about the use of self-monitoring tools for older adults' personal wellness	International Journal of Medical Informatics	Qualitative	HCP (10): nursing experience (8), director of a nursing facility (1), geriatric psychiatrist (1)	Patients (31)	Purposive sampling of HCPs through email lists within professional gerontological/geriatric networks and of older adult participants through contact with an activities coordinator in a local retirement facility	Focus group (in person)	Open coding	Geriatric care	No	Health apps and wearables (consumer technology)	Wellness-related PGHD: Social, spiritual, cognitive and physiological measures	HCPs assumed patients would track data, but they would decide what data is displayed and discussed with patients. There was no further discussion on how to access data.	HCPs were positive about wellness-related PGHD from self-monitoring tools. They found benefits in improving patient-provider communication and educating patients and caregivers. HCPs assumed they controlled selecting PGHD for tracking and what/when to present it to the patient.
Jacomet et al. (2020)[12]	FR	To address whether mHealth serves people living with HIV and their physicians as part of a broader self-managed care service	Médecine et Maladies Infectieuses	Quantitative	HIV care physicians (255)	Patients (287)	Physicians were recruited from 51 clinics who had consultations with people with HIV; patients with HIV were recruited from those clinics	Survey (online)	Descriptive statistics, multivariate analysis	HIV infection	No	Health apps and wearables (consumer technologies)	Various physical and psycological HIV-parameters tracking	Not directly assessed but only 20% of HCP think data integration in PHR is feasible	Health apps are not yet integrated into standard HIV care. However, HCPs see the potential, including in derived PGHD: 40% see apps as helpful for clinical decision support and 37% for monitoring improvements. Only 18% thought apps could improve patient-provider relationships.
Karduck & Chapman-Novakofski (2018)[13]	CA	To identify factors that may be associated with app use by clinicians working in diabetes and weight management patient care settings	Journal of Nutrition Education and Behaviour	Quantitative	Clinicians (719): Registered dietitian nutritionists, registered nurses, certified diabetes educators, board-certified advanced diabetes, advanced practice nurses, doctorates of pharmacy	No	Through electronic mailing lists from four professional groups related to dietetics and diabetes care	Survey (online)	Descriptive statistics, chi-square test and univariate binary logistic regression	Diabetes, obesity	No	Health apps and wearables (consumer technologies)	Diet, physical activity	Not discussed	Most clinicians (62%) recommended smartphone apps to their clients to track diet and physical activity levels. More than 80% of clinicians preferred digital tracking with apps over traditional methods. Discussed barriers include patients' literacy, inaccurate data through app errors and workload on patient.
Kelley et al. (2017)[36]	USA	To understand the perspectives of student health professionals on the usefulness of tracking for assessment, communication and self-care planning for student's mental wellbeing	Proceedings of the 2017 CHI Conference on Human Factors in Computing Systems	Qualitative	Student Health Professionals (14): Psychiatry (9), primary care (2)<br>women's health (2), health promotion (1	Students (297)	HCPs: on campus recruitment not specified; students: social media postings and snowball sampling	Card Sorting Session with HCP (in person)	Card sorting: Think-aloud, inductive iterative thematic analysis, descriptive statistics; Student survey: descriptive statistics, inductive thematic analysis	Mental Health	No	Health apps and wearables (consumer technology)	Various actively and passively collected mental health-related data points were explored in hypothetical scenarios	Hypothetical scenario where HCPs would have access to data prior consultation	Student health professionals perceive access to PGHD as useful depending on data type and case context.
Keogh et al. (2021)[14]	IRE	To understand the experiences and opinions of researchers from academic, industry and clinical contexts in the use of wearable devices to measure gait and physical activity	Journal of NeuroEngineering and Rehabilitation	Qualitative	Researchers (20): biomedical science (2), computer science (1),<br>doctor (4), engineering (3), information technology (1), physiology and/or sport and movement science (4), physiotherapy (5)	No	Purposive, convenience sampling from the Mobilise-D consortium	Interview (video conference platform)	Inductive thematic analysis	Mobility issues	No	Wearables (consumer technology and medical grade)	Gait and physical activity	Access to raw PGHD for research not further specified	Researchers value PGHD from wearables because of its novel insights, which complement traditional data sets. PGHD use requires new protocols. Barriers to use include data management and clear clinical utility.
Kessel et al. (2016)[25]	DE	To investigate the attitude HCPs toward telemedicine, mHealth, and mobile apps in oncology	JMIR	Quantitative	Internal medicine, surgery and other care professionals (108): Resident physicians (24), attending physicians (17), senior physicians (27), heads of department (8), nurses (15), others (17)	No	Convenience sampling through in-hous clinic email distributer	Survey (online)	Descriptive statistics	Cancer	No	Health apps for cancer care	Cancer care-related parameters	Direct integration in the hospital information system (74%, 67/91), export for inspection and analysis via PC (59%, 54/91),<br>or mobile device (52%, 47/91) were highly recommended.<br>Paper-based data provision (24%, 22/91) or email (14%, 13/91)	Most HCPs (84.3%) supported the idea of an oncological app to complement classical treatment, supporting consultation and patient-provider communication with PGHD. Listed helpful features for PGHD collection included side effects, quality of life and others. Most HCPs (93.5%) also supported the use of collected data for scientific research.
Kim et al. (2021)[32]	CA	To explore clinicians and older adults' perceptions of PGHD	JMIR Aging	Mixed methods	HCPs (4): primary care physician (1), nurses (2), physiotherapist (1)	Older adults (5)	Convenience sampling and snowball recruitment via mail to local clinicians and research support groups	Survey, focus groups (not specified	Thematic analysis for interviews, descriptive statistics for survey	Geriatric care	No	Health apps and wearables (consumer technology)	Various PGHD, including weight, blood pressure, blood glucose, diet, medication	Access via decision support systems preferred option to pre-process PGHD and alert	Clinicians evaluated PGHD as useful for monitoring treatment and identifying trends/triggers for older adults. Data reliability, e.g. through noncompliance, was a concern, as well as data privacy/security issues, workload and data overload. Identified useful PGHD included blood glucose, step count, physical activity, sleep, blood pressure, and stress level.
Kong et al. (2020)[15]	USA	To investigate physicians' attitudes towards the adoption of mHealth technologies	Digital Health - Sage	Quantitative	Physicians of 36 medical specialities (186)	No	Via email to HCP university medical school	Survey (online)	Not specified	Not specified	No	Health apps and wearables (consumer technology)	Various PGHD	Direct integration in EHR desired with real-time updates	A majority of physicians see collected biometrics from apps and wearables as useful to promote a healthy lifestyle (68%), track medical treatment (64%), or conduct research (56%). Proof of accuracy and precision (81%) - and the efficient integration of collected data (68%) - preferably directly in EHR - were identified as important improvements.
Lavallee et al. (2020)[16]	USA	To investigate HCPs' perspectives and experiences in PGHD use to understand associated value and barriers	mHealth	Qualitative	HCPs (15)	Healthcare consumers (21), healthcare administrators (5)	Open recruitment and purposive sampling: HCPs were recruited based on previous work by the study team and direct outreach; healthcare consumers were identified through outreach to patient advisory and research networks, recruitment of healthcare administrators not specified	Interview (not specified)	Open coding analysis, followed by inductive coding for sub-themes	Not specified	No	Health apps, wearables and geolocation technologies (consumer technologies)	Mental health and behavioural data, home monitoring of blood glucose; PROMs	Intergration in EHR desired	HCPs see many benefits of using PGHD along the patient journey, including supporting care decisions and improving patient-provider communication and engagement. Barriers to using PGHD are concerns about data validity and lack of integration in clinical workflow.
Nguyen et al. (2019)[26]	AUS	To investigate GPs perspectives of their current and future roles in the use of health apps by their patients and how patient-focused apps affect patient management	Oxford Academic Family Practice	Qualitative	GPs (10)	No	Recruitment through personal network	Interview (in person)	Inductive thematic analysis	Not specified	No	Health apps (consumer technology	Menstrual cycle, blood pressure and blood glucose were mentioned	During consultation preferred, in between visits considered as not feasible	GPs see the benefit of health apps in patient care and PGHD as an additional source of information about a patient. However, apps and PGHD are not yet integrated in clinical practice.

Author (year)	Country	Objective	Journal	Study type	Medical professions (number of participants)	Other participants (excluded from analysis)	Recruitment	Data collection	Data analysis	Disease indication focus	Study part of clinical research program	mHealth technology	Type of PGHD collected or discussed	Provider access to PGHD	Main findings
Nundy et al. (2014)[37]	USA	To explore HCP perceptions of a PGHD report from a text-message-based diabetes self-management program	Journal of Diabetes Science and Technology	Mixed methods	Primary care physicians and endocrinologists (12)	No	HCP with at least one patient enrolled in CareSmarts study were recruited via mail	Survey (in person), interview (in person)	Interviews were analysed with the constant comparative method, with no a priori hypotheses; descriptive statistics for survey	Diabetes	CareSmarts program	App for text-based diabetes monitoring	Diabetes treatment-related information relevant for HCPs, including medication adherence, glucose monitoring	Sending report directly to physician and access through EHR preferred method in preparation for consultation	Only 25% of HCPs felt access to PGHD diabetes report impacted the care they provided. However, 75% would be willing to continue using it. Perceived benefits of PGHD included agenda setting, assessment of self-care, and identification of patient barriers. Concerns were raised about which patients should track and what, data reliability and workflow integration
Osborne et al. (2021)[17]	USA	To identify app content and feature needs from individuals with stroke and traumatic brain injury, caregiver and care provider	British Journal of Occupational Therapy - Sage	Qualitative	Neurorehabilitation therapists (8)	Patients (5), care partners (3)	HCP participants were recruited from the medical centre rehabilitation inpatient and outpatient clinic and one additional School of Health Professions faculty; patient participants were recruited from the medical centre stroke support group or outpatient clinic	Focus group (in person, separate for HCPs and patients)	Thematic analysis	Stroke and traumatic brain injury	No	Health apps	Physical activity, behaviour and related information for disease monitoring	Integration in EHR desired	Therapists favour an app for remote access to PGHD with data integration into the EHR to allow collaboration among HCPs. To manage workload, a dedicated care coordinator should review data first and alert other HCPs accordingly.
Ostherr et al. (2017)[27]	USA	To investigate why and how researchers, health technology start-up companies, and members of the general public interact with and understand the value of PGHD	Big Data & Society - Sage	Qualitative	Behavioural and computational scientists (10)	General public participants: (12), industry stakeholders (10)	Convenience sampling through network with local experts (researcher, industry stakeholder) and at three highly trafficked urban park (general public participants)	Interview (in person or phone)	Inductive analysis	Not specified	No	Health apps and wearables (consumer technology)	Not specified	Not discussed	Research reported difficulties recruiting patients for research that involves them sharing PGHD - which is in great contrast to the finding that members of the general public who are using wearables and/or health apps expressed little concern about sharing health data with the companies that provide the devices or apps. Researchers have concerns about data interpretation and trust in the source of PGHD.
Reading et al. (2018)[38]	USA	To investigate individual patient differences in sustained engagement among individuals with a history of Atrial fibrillation (AF) who are self-monitoring using mHealth technology (iHeart trial)	Applied Clinical Informatics	Qualitative	AF care professionals (8): nurse practitioners (4), physicians (2), research coordinators (2)	Patients (13)	Recruitment of all participants from the iHeart trial	Interview (in person), focus group (in person)	Directed content analysis guided by theory of acceptance and use of technology (UTAUT) approach	Atrial Fibrillation	iHeart trial	AliveCor ECG monitor and app	ECG data	Through a dedicated portal	HCPs see the device's usefulness for patient self-management and medical care. Interaction and feedback from HCPs on PGHD impacted patients' engagement status. HCPs expressed concerns about additional workload and unaligned expectations regarding feedback and tracking burdens for patients.
Saleem et al. (2022)[28]	USA	To assess clinicians perspective on the use of Fitbit PGHD to care for their Veteran patients and to investigate barriers for Veterans to setup and use Fitbits, their perceived value of device features and sharing data with the U.S. Department for Veteran Affairs (VA) (Fitbit pilot program)	Applied Clinical Informatics	Qualitative	Veteran care professionals (16): dieticians (7), physical therapists (2), physicians (2), nurse practitioner (1), sleep medical technologist (1), respiratory therapist (1), nurse (1), licensed practical nurse (1)	Veterans (26)	VA clinicians were recruited via the Fitbit pilot program from four VA medical centres; veteran patients were recruited via mail from the same Fitbit pilot program	Interview (video conference platform)	Frequency of occurrence analysis	Not specified	Fitbit pilot program	Fitbit, data sync app	Fitbit data	Web-based provider platform integration in EHR desired 4/16 HCP felt it would be important to separate PGHD data from EHR data	Veteran clinicians saw the benefit of having Veterans use Fitbits and saw the value of PGHD in the Veterans' care plan, including monitoring progress towards health behaviour goals.
Sanger et al. (2016)[18]	USA	To investigate the tensions between patients' and providers' needs when designing a novel, patient-centred technology – mobile Post-Operative Wound Evaluator (mPOWEr) – that uses PGHD for post-discharge surgical wound monitoring.	Journal of the American Medical Informatics Association	Qualitative	Surgery care professionals (11): Attending physician (4), resident physician (1), nurse practitioners (3), physician assistant (1), clinic nurses (2)	Patients (13), patient advocates (6)	Participants were recruited at two two university-affiliated general surgery clinics having experience with post-discharge surgical site infections	Interview (in person)	Grounded theory analysis	Post-discharge surgical site infection	mPOWEr study	mobile Post-Operative Wound Evaluator app (mPOWER)	Symptoms, photos and communication with provider	Web-based provider dashboard for study purpose, integration in EHR desired	HCPs and patients recognise PGHD as useful in acute, post-surgical care settings. However, disagreements about data collection and feedback expectations cause tensions.
Sarradon-Eck et al. (2021)[19]	FR	To investigate GPs' perception and expectations toward prescription or recommendation of patient-focused mHealth apps or devices	JMIR Mhealth Uhealth	Qualitative	GPs (36)	No	Purposive sampling via professional phone directory and snowball sampling	Interview (in person), focus group (in person)	Grounded theory analysis	Not specified	No	Health apps and wearables (consumer technologies and on prescription)	Not specified	Integration in EHR desired	GPs see health apps as tools to engage patients in their health management. While PGHD are considered valuable as an additional longitudinal data source, the extra workload created by reviewing data not integrated into the EHR and resulting medical liability questions are of concern. GPs were also concerned about overmedicalisation, de-humanisation of the patient-doctor relationship and commodification of patient data.
Volpato et al. (2021)[30]	CH	To explore GPs' perceptions of the role, benefits, risks, challenges, and future development of wearable devices in family medicine	JMIR Mhealth Uhealth	Qualitative	GPs (19)	No	Recruitment of HCPs at a medical conference focused on new technologies in family medicine	Group discussions (in person)	Mind maps	Not specified	No	Health apps and wearables (consumer technology and medical grade)	Not specified	Real-time PGHD transfer discussed, but access was not further specified	GPs were positive about using wearables/apps for remote monitoring (epilepsy and cardiac diseases as examples), supporting self-management and health goals, as well as research. Concerns were related to PGHD quality and validity, lacking clinical evidence for devices, data privacy and security issues, and data workload.
Watt et al. (2019)[31]	UK	To explore HCPs' attitudes toward their patients' use of wearable technology	Digital Health - Sage	Qualitative	HCPs (12): (GPs (4), junior doctors (3), dietician (1), personal trainer/pharma-ceutical technician (1), consultant nurse (1), occupational therapist (1), and physiotherapist (1)	No	Convenience sampling through flyers to GP surgeries and personal contacts	Interview (in person, video conference platform or phone)	Inductive thematic analysis	Not specified	No	Wearables (consumer technology)	Not specified	Not discussed	HCPs saw value in wearables for self-management that could lead to health improvements and reduced costs for the health systems. Concerns were raised about health obsession, distress through tracking and also the intrusion of the patient's most private sphere by accessing PGHD. Another question was who should pay for the devices if patients are supposed to use them.
Wendrich & Krabbenborg (2022)[20]	NL	To investigate HCPs' perspectives on using smartphone apps for digital self-monitoring in multiple sclerosis, particularly focusing on physician-patient communication, healthcare providers respond to self-monitoring data and the role of patient	JMIR Mhealth Uhealth	Qualitative	HCPs (14): Neurologist (4), MS specialist nurses (7), rehabilitation physicians (2), occupational therapists (1)	No	Purposive sampling of HCP affiliated to hospitals that were planning to participate in a MS app pilot project	Interview (in person or phone)	Combination of deductive and inductive thematic content analysis	Multiple sclerosis	No (but HCPs were affiliated with hospitals that were planning to participate in the MS Sherpa app pilot project)	Health apps and wearables MS self-monitoring	MS self-monitoring data	Integration in EHR desired	MS care specialists were willing to use self-monitoring apps and valued the quantitative data complementing patients' narratives. HCPs wanted to control what app is used and what PGHDs are tracked while delegating tasks to patients. Concerns about the workload on patients and emotional burden were raised.

Author (year)	Country	Objective	Journal	Study type	Medical professions (number of participants)	Other participants (excluded from analysis)	Recruitment	Data collection	Data analysis	Disease indication focus	Study part of clinical research program	mHealth technology	Type of PGHD collected or discussed	Provider access to PGHD	Main findings
West et al. (2018)[39]	UK	To investigate HCP perceived barriers to using PGHD across distinct workflows in clinical settings	CHI '18: Proceedings of the 2018 CHI Conference on Human Factors in Computing Systems	Qualitative	HCPs (13): Cardiologists (4), Mental health specialists (2), emergency doctor (1), junior surgeon (1); hospital doctor (1), GP (1), heart failure nurse (1), oncology nurse (1), audiologist (1)	No	Snowball sampling with clear inclusion criteria	Interview (in person, video conference platform or phone), literature review, literature review (excluded from analysis)	Coding with Work Elements Model and thematic analysis of interviews; literature review analysis not further specified	Not specified	No	Health apps and wearables (consumer technologies)	Various PGHD including symptoms, life events, heartrate data, CGM	1) Integration in EHR desired<br>2) Presentation on device or as paper charts (tech savvy people)	HCPs were positive about PGHD use depending on their medical speciality (surgeon less than cardiologist). Perceived barriers to the use of PGHD depend on the specific clinical context.
Wu et al. (2020)[21]	USA	To investigate the current use of PGHD within mental health care with a focus on workflow integration, clinicians' perspectives on PGHD and selection of tools for patients	JMIR Formative Research	Qualitative	HCPs (12): Psychiatrists (7), clinical psychologists (5)	No	Convenience sampling and snowball sampling through co-author contacts; app types were selected based on the significance of those tracking apps and based on findings from the interviews	Interview (not specified), analysis of health app reviews (excluded from analysis)	Coding with Work Elements Model and thematic analysis for interviews; data from user reviews was analysed for app features	Mental health	No	Health apps and wearables (consumer technologies)	Mental health related PGHD including sleep and mood data	Integration in EHR desired	Mental health clinicians reported PGHD collection has always been a part of mental health practice. However, collection and management are not standardised or optimised. PGHD are considered as valuable information, but concerns are raised about data validity, reliability and workflow integration.
Zhu et al. (2017)[22]	USA	To identify enablers and barriers inherent to sharing PGHD for patient-clinician communication and to gain insights into design requirements for future technology interventions	AMIA Annual Symposium Proceedings Archive	Qualitative	HCPs (9): Physical therapist (1), internists (4), primary care physician (1), psychologist (1), paediatric nephrologist (2)	Patients (12)	Purposive sampling: Participants were recruited by word-of-mouth referrals and advertisements in handouts placed in public locations, hospitals, and online (e.g., university mailing lists)	Interview (in person, video conference platform or phone	Qualitative open coding	Not specified	No	Health apps and wearables (consumer technologies)	Not specified	PGHD on device or spreadsheet during consultation; synchronous and asynchronous sharing, e.g. through patient portals, discussed<br><br>Integration of data in patient portals is favoured but needs dedicated staff to pre-process data before HCP reviews it	Various technical, social, and organisational challenges were discussed for using PGHD in clinical practice, including sharing approaches, reimbursement, expectation management, quality of PGHD, and workload.
